# Updates in *Paracoccidioides* Biology and Genetic Advances in Fungus Manipulation

**DOI:** 10.3390/jof7020116

**Published:** 2021-02-04

**Authors:** Alison Felipe Alencar Chaves, Marina Valente Navarro, Yasmin Nascimento de Barros, Rafael Souza Silva, Patricia Xander, Wagner Luiz Batista

**Affiliations:** 1 Department of Microbiology, Immunology and Parasitology, Federal University of São Paulo, São Paulo 04023-062, Brazil; felipealison@gmail.com (A.F.A.C.); marinavnavarro@hotmail.com (M.V.N.); rafael.dsouzas@gmail.com (R.S.S.); 2 Department of Pharmaceutical Sciences, Federal University of São Paulo, Diadema 09913-030, Brazil; barros_yasmin@hotmail.com (Y.N.d.B.); patricia.xander@unifesp.br (P.X.)

**Keywords:** neglected diseases, *Paracoccidioides*, mycosis, dimorphisms, paracoccidioidomycosis

## Abstract

The dimorphic fungi of the *Paracoccidioides* genus are the causative agents of paracoccidioidomycosis (PCM). This disease is endemic in Latin America and primarily affects workers in rural areas. PCM is considered a neglected disease, despite being a disabling disease that has a notable impact on the public health system. *Paracoccidioides* spp. are thermally dimorphic fungi that present infective mycelia at 25 °C and differentiate into pathogenic yeast forms at 37 °C. This transition involves a series of morphological, structural, and metabolic changes which are essential for their survival inside hosts. As a pathogen, the fungus is subjected to several varieties of stress conditions, including the host immune response, which involves the production of reactive nitrogen and oxygen species, thermal stress due to temperature changes during the transition, pH alterations within phagolysosomes, and hypoxia inside granulomas. Over the years, studies focusing on understanding the establishment and development of PCM have been conducted with several limitations due to the low effectiveness of strategies for the genetic manipulation of *Paracoccidioides* spp. This review describes the most relevant biological features of *Paracoccidioides* spp., including aspects of the phylogeny, ecology, stress response, infection, and evasion mechanisms of the fungus. We also discuss the genetic aspects and difficulties of fungal manipulation, and, finally, describe the advances in molecular biology that may be employed in molecular research on this fungus in the future.

## 1. Introduction

### 1.1. Paracoccidioides and Paracoccidioidomycosis

*Paracoccidioides* spp. is the aetiologic agent of paracoccidioidomycosis (PCM), a systemic mycosis that mainly affects workers in rural areas and causes some degree of disability for working-age people. In 1908, Adolpho Lutz described the causative agent of PCM in the oral lesions and cervical lymph nodes of two patients. By that time, the disease and its agent received several names, until in 1930 Floriano Almeida finally proposed the name *Paracoccidioides brasiliensis* [1]. More recently, genomics analyses have identified the existence of cryptic species within the *Paracoccidioides* genus and new species have been proposed [2,3].

**Figure 1:**
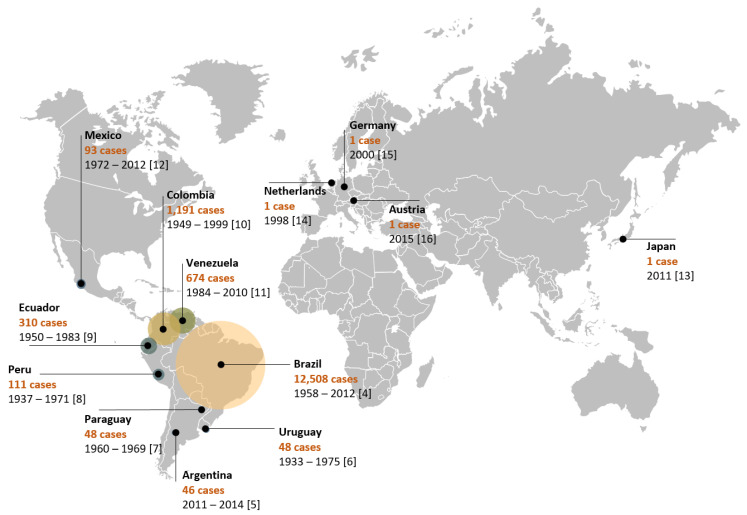
Epidemiological distribution of PCM cases throughout the world. The Netherlands, Germany, Austria, and Japan have imported cases. Legends show the name of the country and the number of cases identified through the period mentioned in each study.

The disease is widely distributed in Latin American countries, such as Brazil [4], Argentina [5], Uruguay [6], Paraguay [7], Peru [8], Ecuador [9], Colombia [10], Venezuela [11], and Mexico [12]. However, there are reports of imported cases in such countries as Japan [13], the Netherlands [14], Germany [15], and Austria [16] (Figure 1). Due to the reduced lethality and restricted geographic distribution of the etiological agent of PCM, public health agencies have largely neglected the disease. PCM is geographically limited to Latin American countries and the highest number of reported cases are concentrated in Brazil (80%). Although Brazil reports more cases than any other country (Figure 1), these numbers are probably underestimated, given that until February 2020 PCM was not a notifiable disease, although there is currently a recommendation for reporting cases as proven or probable [17]. It is expected that with this change, the data regarding epidemiology will soon be more accurate.

### 1.2. Paracoccidioidomycosis: Disease, Diagnosis and Treatment

PCM is a granulomatous disease that usually presents in two distinct forms: acute or subacute and chronic forms. The acute or subacute form is responsible for 5–25% of cases and is characterized by rapid dissemination to the reticuloendothelial system. In the chronic form (74–96% of cases), the disease progresses slowly and may take months to years to become apparent, and pulmonary manifestations are present in more than 90% of cases [17,18]. In addition, the chronic form can manifest as unifocal or multifocal. In the unifocal form (25% of cases), the lungs and, rarely, other sites are the only organs involved [19]. In the multifocal form, along the lungs, the main sites involved are the oral mucosa, skin, lymph nodes, and adrenal glands and, to a lesser extent, the central nervous system, bones, genitals, and blood vessels. The residual form, observed less frequently, is expressed by sequelae left after treatment [17].

The clinical manifestations of PCM can be related both to characteristics inherent to the pathogen and to the host’s immune response profile. The analysis of the adaptive immune response in different clinical spectra of PCM has contributed to a better understanding of the disease evolution. The presence of the T helper 2 (Th2)/Th9 profile with a high production of cytokines IL-4, IL-5, IL-9, IL-10, TGF-β, IL-27; the polyclonal activation of B cells; the production of large amounts of specific IgG4, IgA, and IgE; and a low production of IFN-γ and TNF-α is related to the acute form of PCM with the fungus spreading to different organs and systems [20,21]. On the other hand, the prevalence of Th17 and Th22 with the production of the cytokines IFN-γ, TNF-α, and IL-2; varying amounts of IL-10 and IL-4; and the presence of high levels of specific IgG1 antibodies are immunological characteristics of the chronic form [20,21]. Asymptomatic and mild chronic forms have a Th1 profile, while the severe chronic form may have a predominance of Th2 response [21]. Interestingly, mice infected with different *P. brasiliensis* genotypes showed differences in their immune response profile, suggesting that factors of the pathogen may also contribute to the different activation profiles [22]. The review of innate and adaptive host immunity to *Paracoccidioides* was not the purpose of this work. These aspects are widely covered in another review by Calich et al., 2019 [23].

PCM is rarely observed in children and young people and is more common in men aged 30 years or more. The susceptibility of men and women does not differ notably; the ratio of men who develop the disease to women is 22:1 [17]. This difference has been attributed to the presence of high levels of endogenous estrogens in women that act as binding proteins in the fungal cytosol, inhibiting the transition to the pathogenic phase [24,25,26]. In fact, pre-adolescent girls, women in menopause, and women suffering from hormonal disorders may manifest the disease, given their decrease in 17β-estradiol [27]. Although the relationship between the presence of estrogens and the clinical manifestation of the disease can be explained by epidemiological data and by experiments inhibiting the mycelium–yeast transition, the understanding of the complex relationship between steroid hormones and the immune system is rapidly developing and may help to elucidate the differing disease responses between women and men [28]. Therefore, the mechanisms underlying estrogen protection against PCM warrant further clarification.

The clinical diagnosis of PMC remains a challenge, given the ability of the fungus to mimic the most diverse pathological conditions, such as tuberculosis, leprosy, histoplasmosis, coccidioidomycosis, blastomycosis, leishmaniasis, and syphilis, as well as non-infectious diseases, including bone tissue neoplasia, oral and pharyngeal cancer, cholangiocarcinoma, hypercalcemia, Crohn’s disease, Hodgkin’s lymphoma, icteric syndromes, sarcoidosis, and others [16,17,29,30,31,32]. Such variability of clinical presentations makes it necessary to include PCM among the differential diagnoses for patients who live in or have visited endemic areas, even for patients with symptoms that are not suggestive of PCM. Particular attention should be directed to patients with immunosuppression, such as patients with HIV, cancer, transplants, and autoimmune diseases. There are at least nine reported cases of renal transplant patients who developed PCM [33]. Data in this population remain scarce, and the differential diagnosis becomes even more important for implementing the correct treatment.

The diagnosis of PCM is based on clinical and laboratory findings. In the acute form of the disease, skin lesions are frequently observed. On the other hand, in the chronic form the lungs are more often affected. In these cases, in endemic areas chest radiography may present bilateral and symmetrical opacities in the midline of the lungs, a finding known as butterfly wings [34]. The laboratory diagnosis of PCM must be based on the direct mycological examination of biological materials, such as sputum, bronchial lavage, lesion scraping, lymph node aspiration, or biopsy samples, in which the typical morphology of the fungus can be visualized. Under a light microscope, small chains of blastoconidia or single budding cells can be observed. Histopathologically stained preparations can demonstrate multiple budding yeasts, preferably within granulomas. Although the isolation of the agent is not always possible, the biological material can be cultured at 25–30 °C to finalize the diagnosis [19]. Serological tests are utilized to facilitate the diagnosis, to monitor the evolution of the disease, and to measure the response of the patient to the treatment of this mycosis [35]. Radial immunodiffusion using yeast phase culture filtrate is the most frequently utilized method, presenting a high sensitivity and specificity [35]. Other serological methods, such as immunoblotting and ELISA [35], have been developed for patient monitoring. Molecular methods using PCR have also been proposed to facilitate the definitive diagnosis of the fungus but have not been adopted for use in routine laboratory tests [35]. The standardization of diagnostic techniques among clinical laboratories is still a challenge. The identification of new antigens and the development of better diagnostic methodologies can contribute to the correct identification and better monitoring of PCM patients.

Regarding the therapy of PCM, the first-line treatment is itraconazole, a drug of choice for the treatment of mild and moderate forms of PCM within a time period of 9–18 months. However, given the interactions between itraconazole and a wide range of drugs often used by patients with comorbidities, cotrimoxazole (18–24 months) can be used as a second-choice line of treatment. Sulfamethoxazole is indicated when associated with trimethoprim (an association known as cotrimoxazole). Among the advantages of this association are the low cost, good tolerance, safety for prolonged use [19], and good penetration in the central nervous system [36]. Cotrimoxazole is used in patients with mild to moderate forms of PCM and neuroparacoccidioidomycosis [36]. Although the side effects of itraconazole and cotrimoxazole (e.g., nausea, vomiting, and diarrhea) can make treatment difficult, overall both drugs are well tolerated by patients with PCM.

In severe and disseminated forms, the drug of choice is amphotericin B, and the patient needs to maintain the treatment with itraconazole or cotrimoxazole for an extended period [17]. Despite the effectiveness of amphotericin B, it produces several toxic effects in the host, including acute symptoms, such as nausea, vomiting, fever, hyper- and hypotension, and hypoxia, in addition to chronic nephrotoxicity. New formulations, such as amphotericin B lipid complex, liposomal amphotericin B, and amphotericin B colloidal dispersion, have yielded better tissue distribution and less toxicity [36].

### 1.3. Paracoccidioides Phylogeny and Ecology

PCM was initially recognized as being caused only by the fungus *P. brasiliensis* [37]. After 75 years, phylogenetic studies demonstrated the need to separate the etiological agent in at least three distinct phylogenetic species (PSs)—namely, S1 (38 isolates), PS2 (6 isolates), and PS3 (21 isolates) [2]. This was because the *Paracoccidioides* genus seems to contain several cryptic species. Cryptic species are those that cannot be easily distinguished based on morphology but which form distinct phylogenetic lineages based on molecular markers. Although researchers recognized the need for separating identified PSs, for years, the isolates continued to receive names related to *P. brasiliensis*, such as Pb01, Pb03, Pb18, and Pb339, among others. However, there remained a set of isolates, including Pb01, that differed from the proposed PSs. Soon, isolate Pb01 and others (an additional 16 isolates) were recognized as a new species, P. lutzii [3].

This set of changes in the classification of *Paracoccidioides* isolates occurs frequently among organisms recognized as cryptic species [38]. At present, it is believed that PCM is caused by fungi of the genus *Paracoccidioides* [39], which is composed of at least five species (*P. brasiliensis*, *P. lutzii*, *P. americana*, *P. restrepiensis*, and *P. venezuelensis*) [40]. Despite the great genetic variability among these species, there do not seem to be important differences in the clinical findings when comparing *P. brasiliensis* with *P. americana* [41] or *P. lutzii* [42], for instance. Currently, the genus *Paracoccidioides* is taxonomically classified as Eukaryota of the kingdom Fungi, phylum Ascomycota, subphylum Pezizomycotina, and family *Ajellomycetaceae*. Additionally, part of this family includes the fungi *Histoplasma capsulatum*, *Blastomyces dermatitidis*, and *Coccidioides immitis* [1].

Although overlooking the fact that fungi of the genus *Paracoccidioides* are organized in cryptic species, the recognition of the existence of isolates with different degrees of virulence, as in the comparisons between Pb18 (considered virulent) and Pb265 (considered of lower virulence), enabled the establishment of an animal model to study the disease [43,44]. Today, however, it is known that even isolated Pb18, considered virulent, can become attenuated after successive culture passages [45]. This model of attenuation and virulence recovery through inoculation in animals enables the study of virulent and attenuated fungi of the same genetic background.

The *Paracoccidioides* fungi are found in soil, which may be the primary source of infection for both humans and wildlife, given the lack of evidence of transmission between hosts or reservoirs and hosts [4]. Natural infections have been described in some wild and domestic animals [46], and a *Paracoccidioides* fungus was isolated from two armadillo species, *Dasypus novemcinctus L*. and *Cabassous centralis* [47]. These findings suggest that the armadillo is an important natural reservoir of *Paracoccidioides* [48], and that the same animal can carry multiple species of the fungus [49]. Close contact with soil and animal digging habits enable the dissemination of fungal propagules [47]. Furthermore, the armadillo appears to be highly susceptible to infections [50]. *Paracoccidioides* fungi have been isolated from infected animals in various states, such as Pará, São Paulo, and Minas Gerais [39]. Approximately 75% to 100% of all animals caught in endemic areas are favorable for PCM [47]. The permissiveness of infectivity in several mammalian hosts can help to explain the spread of the *Paracoccidioides* fungi over extensive areas. In addition, the great variability observed among the various isolates may be due to environmental pressure and/or to immune responses from different animal species infected by fungi. More in-depth research on these topics may help to elucidate the genetic variability and ecology of *Paracoccidioides*.

In this review, we aimed to summarize the major findings in the field of *Paracocccidioides* biology as well as its genetic aspects. Next, we describe and detail the findings related to dimorphism, stress response, infection, and evasion mechanisms and point out the difficulty and advances in the genetic manipulation of this fungus. Understanding these aspects is necessary to advance in identifying virulence factors, diagnostic markers, new pharmacological targets, and therapeutic strategies.

## 2. Fungal Biology

### 2.1. Dimorphic Transition

**Figure 2:**
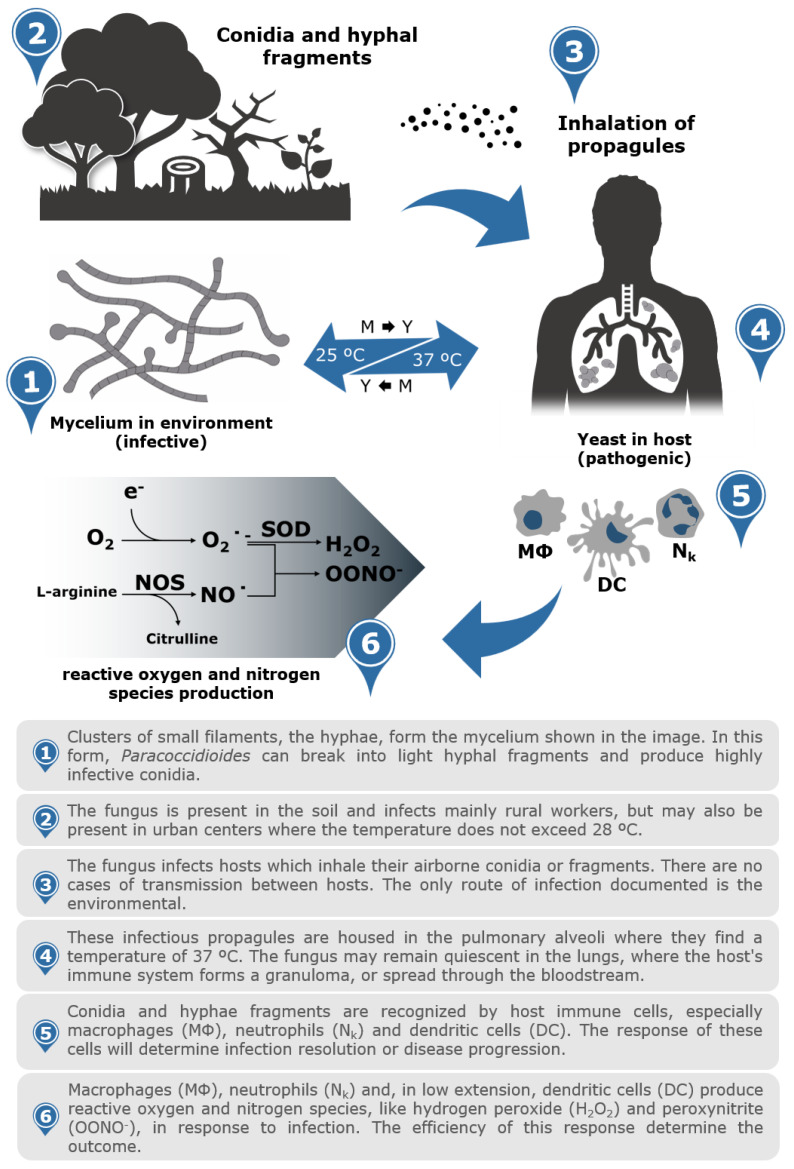
Infographic of *Paracoccidioides* species life cycle from the environment to the host. The saprophytic form of *Paracoccidioides* spp. are found in the soil as mycelium (25 °C). Conidia and hyphal fragments inhaled by mammalian hosts are the primary sources of infection. The propagules are inhaled and established in the lungs, which, at body temperature (37 °C), initiate the dimorphic transition to pathogenic yeast form. The immune cells present within the host, such as macrophages, neutrophils, and dendritic cells, recognize the pathogen and trigger defence mechanisms such as the production of reactive nitrogen and oxygen species (RNS/ROS).

The fungi of the genus *Paracoccidioides* present different morphologies when subjected to different temperatures. This feature is called thermodependent dimorphism, and temperature change is a critical event in the disease of these fungi, since strains that do not differentiate into yeast cells are avirulent [51]. When these fungi are grown at room temperature (between 19 and 26 °C), they present as mycelia, the infective form. On the other hand, when subjected to temperatures close to those of the mammalian body (36–37 °C), the hyphae differentiate into yeasts, marking the pathogenic phase. The transition from mycelia to yeast cells can be induced in vitro by changes in the incubation temperature from 25 to 37 °C [18,52] (Figure 2). The mycelial form presents tangles with long filaments of septate and branched hyphae, which are thin and hyaline. Yeasts are multinucleated and of varying sizes (4 to 50 μm), and they have a spherical, oval, or elongated shape and multiple buds. When yeasts are cultured in solid medium, they present elevated colonies with irregular borders, cerebriform appearance, and cream coloring, becoming darkened with ageing [53].

Although the dimorphic nature of *Paracoccidioides* spp. has been well-known for decades, the molecular regulators and effectors of dimorphism have not been fully elucidated. It is known that in several pathogenic fungi, the cAMP/PKA pathway controls morphology and pathogenicity and that organisms can respond quickly to environmental changes through this pathway. A study performed in *P. lutzii* demonstrated that when PKA activity is inhibited, the transition from mycelium to yeast is compromised [54]. Additionally, there is an increase in cyclic adenosine 3′,5′ monophosphate (cAMP) in the process of dimorphic transition in *P. brasiliensis* [55]. Exogenous cAMP inhibits the *P. brasiliensis* yeast-to-mycelium (Y–M) transition, thereby maintaining the pathogenic yeast form [56]. This situation differs in *Candida albicans*, in which the Y–M transition is controlled by cAMP, and exogenous cAMP stimulates pseudo-hyphae that may be able to invade mammalian cells [57].

A number of initial studies identified genes and proteins involved in the mycelium–yeast transition and made an interesting connection between yeast morphology and the expression of virulence genes [22,58,59,60,61,62,63,64]. However, few genes have been identified as phase-specific in *Paracoccidioides* spp. yeasts [22]. Among the markers of the pathogenic phase are α-1,3-glucan synthase (*AGS1*), which is responsible for the synthesis of cell wall sugars [51], and dimorphism-regulating histidine kinase (*DRK1*), which is implicated in the control of dimorphism [65]. In addition to the search for specific markers for the pathogenic phase, several studies have attempted to determine a set of proteins that are representative of each of the phases, as well as the transition [60,63]. In *P. lutzii* [66], six antigens preferentially synthesized in the yeast phase of the fungus were identified: catalase, fructose 1,6-bisphosphate aldolase, glyceraldehyde-3-phosphate dehydrogenase (GAPDH), malate dehydrogenase, and triosephosphate isomerase. Another study determined the profile of secreted proteins in the pathogenic and environmental phases [64].

Heat shock proteins (HSPs) are also widely regulated during the phase transition because dimorphism is conditioned by temperature changes [59]. Although temperature changes trigger the differentiation process, GTPase Ras seems to be a fundamental component in the maintenance of cells in the yeast phase. Indeed, the inhibition of Ras farnesylation induces filamentation in yeasts of *Paracoccidioides* in a temperature-independent manner [67]. On the other hand, the protein paracoccin (PCN) appears to be fundamental in the process of the reversion of yeasts to mycelium [68].

Some proteins involved in the dimorphic transition of *P. brasiliensis* were identified in a study with microRNAs. Genes encoding dcr-1 were predominant in yeast cells, while dcr-2 and aug-2 exhibited increased expression in the mycelial phase. Thioredoxin, glutathione *S*-transferase, and chaperone hsp12 protein production is reduced when fungi are in the form of mycelium or are in the mycelium–yeast transition because they are the targets of microRNAs [69].

The morphological change in *Paracoccidioides* is accompanied by changes in the cell wall composition [69], including the migration and reorganization of membrane lipids, especially glycosphingolipids (GLSs) [70]. Indeed, yeasts have a greater amount of chitin than the hyphae of *Paracoccidioides* [71], which is explained by the increase in the expression of the enzyme chitinase CTS2 in the hyphae and the reduced expression in yeasts of this fungus [60]. This change in the chitin amount in the cell wall may be critical to fungal escape mechanisms [72]. Understanding the steps of morphological transition and the molecular mechanisms involved in this process, both those related to cell metabolism and those involved in important changes in the composition and structure of the cell wall, may help to identify useful molecular targets for treating and preventing PCM development.

### 2.2. Cell Wall

**Figure 3:**
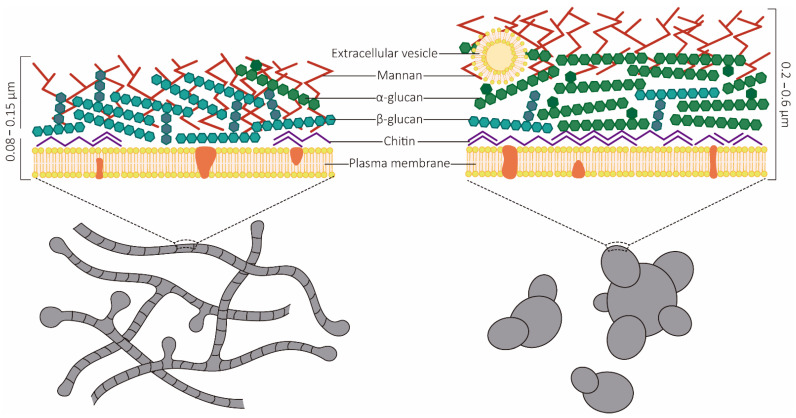
Schematic representation of the *Paracoccidioides* species cell wall. The ability to perform the dimorphic transition is an essential event for fungal establishment in the host system. This alteration is involved in major changes in the cell wall composition. The thickness of the filamentous form is approximately 0.08 to 0.15 μm, thinner than that of the yeast form, which is approximately 0.2 to 0.6 μm. The cell wall of the filamentous form is composed mainly of polysaccharides, such as β-glucan (most abundant), chitin, and mannan (less abundant). The yeast from the cell wall is constituted mainly of α-glucan and chitin (most abundant) and other polysaccharides, such as mannan. In general, the cell walls of both forms contain smaller proportions of amino acids, lipids, and proteins. The predominance of distinct O-glycosidic bond polysaccharides is essential for host adaptation, as β-glucans are recognized by the dectin-1 receptors of phagocytic cells.

The morphological switch from the mycelia to the yeast form is an essential event for disease development and host establishment. This event is associated with major morphological alterations regarding cell wall architecture. The cell wall is thicker in yeasts (0.2–0.6 µm) than in hyphae (0.08–0.15 µm), but in both phases there is an outer layer and an inner layer [73]. The cell walls of *Paracoccidioides* spp. are composed mainly of polysaccharides [73] but also of proteins, lipids, and melanin [74]. The main polysaccharides associated with cell wall structural integrity are β-1,4-linked homopolymers of N-acetylglucosamine (chitin) and glucan [75]. Yeast cell wall composition presents important differences in relation to hyphae. In yeasts, the cell wall is composed of carbohydrates (81%), mainly glucose (38%) and N-acetylglucosamine (43%), with small proportions of amino acids (10%) and lipids (11%). On the other hand, in mycelia 51% of the cell wall contains carbohydrates (38% glucose and 13% N-acetylglucosamine), 33% amino acids, and 8% lipids [73] (Figure 3).

Chitin is largely responsible for cell wall structural integrity, and in mycelia it is less abundant than glucan polysaccharides. However, in the yeast form chitin contents represent almost half of the wall total dry weight [71]. In *P. brasiliensis,* there are five characterized genes encoding chitin synthase enzymes—namely, *CHS1*, *CHS2*, *CHS3*, *CHS4*, and *CHS5* [71,73]. These genes are differentially expressed during fungal dimorphism [71].

One of the most important proteins in the synthesis of the fungal cell wall is glucan synthase [76]. The phenotypic change of fungi entails alterations in the fungal cell wall composition, with a predominance of β-1,3-glucan and β-1,6-glucan and carbohydrates being observed in the mycelial form. In contrast, in the yeast form there is a prevalence of α-1,3-glucan and chitin [77]. Changes may occur in the amount and spatial arrangement of these polysaccharides [73], which ensure the survival of the fungi in the host. The α-1,3-glucan correlates with the degree of virulence [78], possibly by the concealment of β-glucans, which are recognized by the dectin-1 receptors of phagocytic cells [79]. This mechanism facilitates the evasion of the immune response of the host [80]. Indeed, differences in the ability of dendritic cells to phagocytose yeasts from different *Paracoccidioides* isolates have been reported, and this phenomenon was attributed to variations in the cell wall glucan composition [81] (Figure 3).

The proteins present in the fungal cell wall constitute 3–20% of the dry weight and play roles in the structure, organization, and physiology of this component. Surface proteins perform relevant functions, such as promoting adhesion and protecting against phagocytes and components that can damage cells [82]. In addition, a number of these proteins display moonlighting functions, meaning that the proteins have a primary enzymatic function but also have acquired secondary non-enzymatic roles. In this group, proteins involved in glycolytic enzymes, such as enolase, glyceraldehyde-3-phosphate dehydrogenase, fructose 1,6-bisphosphate aldolase, triosephosphate isomerase, cellular signalling protein 14-3-3, and heat shock protein, were detected [68,73]. The PCN protein is another important component located in the cell wall. The absence of the PCN protein appears to facilitate yeast phagocytosis [69]; on the other hand, neutrophils stimulated with a recombinant form of PCN produce more reactive oxygen species (ROS) and become more efficient in eliminating the fungus [83]. In proteomic studies, several proteins were identified in the yeast cell wall, including some antioxidants, such as catalase B, thioredoxin reductase, and nitroreductase, which might play a role in host–pathogen interactions. Most of the identified proteins (approximately 80%) have been classified as secreted [84], and some of them may be involved in the escape of *Paracoccidioides* from the immune response by subverting the activation of immune cells and contributing to the pathogenesis of PCM.

### 2.3. Infection and Evasion Mechanisms

Host–parasite interactions are complex events in which the host is under pressure to develop resistance while the parasite attempts to escape and adapt to the host’s immune response and thus survive in the host environment [85]. Infection with *Paracoccidioides* spp. occurs after the inhalation of conidia or fragments of hyphae. To develop the disease, the conidia housed in the pulmonary alveoli must differentiate into yeasts. The change from infecting to pathogenic phase relies on the increase in temperature [86]. This sudden environmental change associated with infection implies the ability to adapt quickly to survive and invade the host. This phase transition (from mycelium to yeast) is an essential event in fungal biology and results in the expression of the virulence factors necessary to establish the infection [87] (Figure 2).

Virulence factors, in turn, are elements that increase the ability of pathogens to invade, replicate, and persist in the host [88]. The pathogen uses a large repertoire of surface molecules, specifically adhesins, that can bind to the extracellular matrix (ECM) of various cell types in the host. This interaction with the ECM has been correlated with the processes of adhesion and invasion. The ECM is composed mainly of collagen; elastin fibres; glycosaminoglycans (GAGS); proteoglycans (PG); fibronectin; laminin; heparan sulphate; nidogen/entactin; hyaluronate; chondroitin sulphate; and collagens of subtypes I, III, IV, and V [89]. *Paracoccidioides* spp. recognizes several of the components of the ECM—mainly, fibronectin, laminin, plasminogen, and types I and IV collagen [90]. Gp43 proteins and the glycolytic pathway (such as GAPDH and triosephosphate isomerase enzymes) have been described as capable of binding to ECM components, such as laminin and fibronectin [91,92,93,94]. Enolase, fructose 1,6-bisphosphate aldolase, and 14-3-3 protein also showed adhesion properties [95,96], binding to fibronectin and plasminogen, thereby leading to the degradation of the extracellular matrix and facilitating tissue invasion by the fungus [97,98]. The 14-3-3 protein demonstrated the ability to adhere to A549 cells (pulmonary adenocarcinoma cell line) [99].

Some differences concerning the degree of adhesion were observed for *Paracoccidioides* spp. regarding how they enter different cell types. These differences may be related to changes in the composition of the cell wall [77]. Hanna et al. (2000) observed differences in the adhesion capacity of four *P. brasiliensis* strains to Vero cells [100]. Oliveira et al. (2015) observed that *P. brasiliensis* and *P. lutzii* present differences in their capacity of adhesion to pneumocytes [101]. These data support the fact that adhesion and virulence are closely related in *Paracoccidioides* spp. and reinforce the importance of adhesion in the infection process of these fungi.

Fungal adhesion is critical for colonization, leading to the invasion and damage of host tissue. An important component of tissue invasion is the expression and secretion of proteases, which are virulence factors in fungal infections. *P. brasiliensis* showed 53 ORFs encoding proteases, including proteasome subunits, aspartyl, cysteine, metallo-, and serine proteases [102]. However, only a few *Paracoccidioides* proteases have been characterized over the last two decades; these proteases include serine protease and aspartyl proteases (PbSap) [103,104,105,106]. PbSap is a secreted protease that has been reported in culture supernatants [103,104], and its expression increased in the highly virulent isolate Pb18 and decreased after several generations in culture [104]. In addition, a vaccination strategy using a recombinant version of PbSap was able to confer some level of protection against the lung parenchyma lesions inflicted by *P. brasiliensis* in a PCM experimental model [104]. Serine protease has also been identified in *Paracoccidioides* culture supernatants under nitrogen starvation conditions, thereby indicating the potential function of this protein in fungal nitrogen acquisition [105]. This serine protease expression is induced in yeast cells infecting murine macrophages [105] and during the incubation of yeast cells with human plasma [107], thereby suggesting that the protein plays a putative role in PCM pathogenesis.

In addition to protease secretion, melanin is also produced as a putative factor that contributes to fungal protection in the host hostile environment. Melanin is a biopolymer produced in several organisms, and, in fungi is commonly associated with protection against several stress conditions [108]. Synthesis can occur by two distinct pathways: through he polysaccharide-synthetase pathway with endogenous substrates, and through phenoloxidases or laccases pathways using phenolic compounds such as l-3,4-dihydroxyphenylalanine (l-DOPA) [109]. *Paracoccidioides* isolates have been shown to produce melanin in yeast and hyphae [109], this pigment is associated with the pathogenesis of PCM [109,110,111]. *P. brasiliensis* melanized yeast cells are more resistant to attack by the oxidative/nitrosative stress of murine macrophages, show greater resistance to phagocytosis, and are less susceptible to antifungal drugs [109,110,111].

### 2.4. Extracellular Vesicles (EVs)

**Figure 4:**
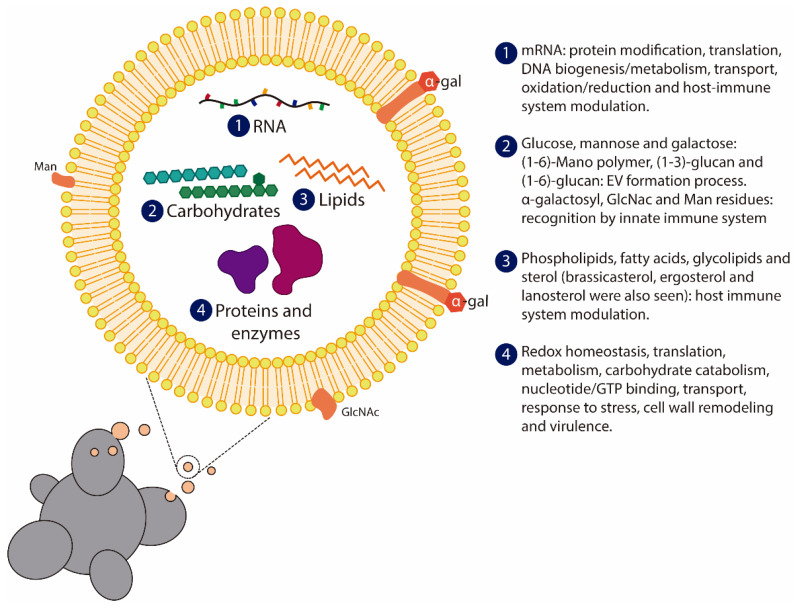
Representation of *Paracoccidioides* spp. extracellular vesicles (EV). Several studies regarding proteomics, lipidomics, transcriptomics, and general EVs composition were performed in order to characterize *Paracoccidioides* spp. EVs composition and content. The secretory pathway can occur by conventional and unconventional release, however these mechanisms are not yet fully characterized in *Paracoccidioides* spp. The content and composition of EVs are distributed among (**1**) RNA (mRNAs, non-coding RNAs, small RNAs, and interference-RNAs); (**2**) carbohydrates (glucose, mannose, and galactose), including N-Acetylglucosamine (GlcNAc) and mannan residues in EVs surface; (**3**) lipids (brassicasterol, ergosterol and lanosterol); and (**4**) proteins and enzymes involved with a myriad of signaling pathways and host immune system modulation.

EVs are membranous vesicles released by eukaryotic and prokaryotic cells, and they play important roles in intercellular communication. These vesicles can carry different molecules, including proteins, lipids, and nucleic acids (Figure 4), between cells within one organism or between organisms in a cross-kingdom interaction, such as in host–pathogen cross-talk [112]. In fungi, EVs are also involved in the transport of macromolecules across the cell wall [113]. Various reports have described the release of EVs by several species of pathogenic fungi in both yeasts and filamentous fungi [114,115,116,117,118,119,120]. Conventional and unconventional secretory pathways have been proposed for the release of EVs by fungi. Although the mechanisms by which EVs are released are not fully understood to date, studies with mutants have demonstrated the importance of some proteins in the production and release of EVs by fungi [114,115,116]. Additionally, the passing of EVs through the cell wall is still under investigation. The presence of channel guides, remodeling enzymes, or turgor pressure are the main hypotheses to explain the passage of EVs by the fungal cell wall [121].

Studies to better characterize the content of EVs released by fungi have contributed to a better understanding of the biogenesis, release, and biological functions of these EVs. In *Paracoccidioides*, the presence of immunogenic α-linked galactosyl epitopes in *P. brasiliensis* EVs was identified [118]. Subsequently, reports identified the contents of carbohydrates, proteins, lipids and RNA in *Paracoccidioides* EVs [122,123,124,125]. Analysis of carbohydrate composition identified small amounts of (1-6)-mano polymer, (1-3)-glucan and (1-6)-glucan in EVs from *Paracoccidioides* [124] (Figure 4). The presence of the cell wall α-1,3-glucan synthase MOK1-like in *P. brasiliensis* EVs suggests a potential contribution to remodeling the fungal cell wall [123]. GADPH, enolase, and gp43 are proteins located in the cell wall that were also observed in *P. brasiliensis* EVs [122]. These molecules have several biological functions in fungal metabolism and the fungal–host relationship [86,92,95]. Thus, analysis of the *P. brasiliensis* EV proteome helped to elucidate protein content and contributed to uncovering the biological functions of these EVs.

The heterogeneity and diversity of EVs released by the *Paracoccidioides* genus were highlighted by analysis of the content of RNAs in the EVs of *P. brasiliensis* and *P. lutzii* (Figure 4) [125]. The presence of exclusive RNA sequences in each species confirms the diversity in EV composition and suggests that fungus–host interactions can be affected by different RNA EV compositions [125].

Notably, a great number of virulence factors are also associated with fungal EVs [111]. However, few functional studies have been performed with *Paracoccidioides* EVs. It was demonstrated that EVs released by *P. brasiliensis* induced murine macrophage polarization to an M1 profile in vitro [126], and macrophage stimulation with EVs led to an increase in the fungicidal activity of these cells [126]. A mammalian lectin microarray assay showed that carbohydrate residues present on the EV surface were recognized by DC-SIGN (a dendritic cell-specific intercellular adhesion molecule-3-grabbing nonintegrin), suggesting that EVs can stimulate the innate host immune response [124]. In addition, after coculturing with *P. brasiliensis* in the Transwell system, dendritic cells modulated the gene expression of several genes related to the immune response [124]. However, functional studies are needed to better assess the mechanisms of EV interactions with host cells. Although these findings are highly speculative, the presence of RNAs and other molecules in the *P. brasiliensis* EV ligands of expressed receptors may partly explain these effects. Additional studies to better understand the direct and indirect effects of *P. brasiliensis* EVs on immune cells and in the immune response may help to elucidate the mechanisms by which the fungus interacts with the host. This knowledge can be applied for new perspectives on the diagnosis, prognosis, and treatment of PCM in the future.

### 2.5. Stress Response

#### 2.5.1. Oxidative and Nitrosative Stress

Alveolar macrophages (AMs) play important roles in developing appropriate immune responses to contain and eliminate inhaled pathogens, such as infective propagules of *Paracoccidioides* [127] (Figure 2). AM and other immune cells recognize pathogen-associated molecular patterns (PAMPs) expressed on fungal cell walls by receptors present on the surface of the cell membrane and in the intracellular compartment [128]. These sensors activate immune cells and trigger several processes that can lead to phagocytosis, the secretion of chemokines and cytokines, and microbicidal species production [128]. Reactive oxygen and nitrogen species (ROS/RNS) released by activated macrophages and/or by other activated immune cells have antifungal properties (Figure 2). However, the characterization of the machinery that allows the adaptation of fungal cells to an environment with nitrosative and oxidative stress may bring new perspectives on host–parasite interactions [129].

Several genes and signaling pathways are regulated by the intracellular redox state [130]. High concentrations of ROS/RNS can lead to molecular damage and deleterious reactions [131]. However, low concentrations of ROS and RNS can benefit *P. brasiliensis*, inducing a proliferative response in fungi [132]. Even in oxidative and nitrosative stress, the fungus can survive through a broad and efficient repertoire of detoxification enzymes, such as catalases; superoxide dismutases (SOD); peroxidases and thioredoxins [133,134]; enzymatic systems, such as glutathione and cytochrome c peroxidase [135]; and classical signaling pathways, such as MAPK Hog1 and GTPase Ras [136], Hsp90 and calcineurin [22,137].

The ability to survive and proliferate in an unbalanced redox environment is closely related to fungal virulence. Using a mass spectrometry approach, proteins involved in the oxidative stress response were identified as differentially expressed in the same isolate of *P. brasiliensis* with different virulence levels acquired through successive passages in an animal model [45]. Gene expression and enzymatic activity showed the distinct responses of antioxidant enzymes between virulent and attenuated isolates. Notably, the *P. brasiliensis* attenuated isolate recovered the virulent phenotype and the antioxidant repertoire after animal passages [138].

Proteomic studies of oxidative stress-based models have demonstrated that different levels of ROS induce distinct phosphorylation patterns at different sites, determining the kinase activities and phosphatase regulation involved in DNA processing and cell cycle control (low levels of H_2_O_2_) or cell survival response (high levels of H_2_O_2_) [139]. Higher levels of ROS lead to the expressive activation of antioxidant enzymes and metabolic alterations. The NAD(P)H production pathway was activated to minimize the oxidative effects caused by peroxide treatment, and metabolism shifted the production of glucose by gluconeogenesis to amino acid synthesis to produce molecules that are important for the oxidative response [133].

Nitric oxide (NO) is also produced as the immune system attempts to eliminate pathogens under nitrosative stress. This molecule can react with several external and intracellular targets [140]. It has been demonstrated that RNS produced by immune cells is associated with the inhibition of dimorphic transition [141] and induces decreased expression of proteins related to the mitochondrial electron transport chain, proteins related to DNA damage and cell survival [142]. Additionally, NO can induce posttranslational modifications, such as nitration and *S*-nitrosylation [143], thereby influencing protein function. Recently, a study detected S-nitrosylated proteins after submitting *P. brasiliensis* to low and high concentrations of NO. Several proteins involved in the cellular cycle and growth were identified in *P. brasiliensis* treated with low NO concentrations [144]. On the other hand, at high NO levels, *S*-nitrosylated proteins involved in the cell wall integrity (CWI) pathway and amino acid and folic acid metabolism were identified, which may represent molecular targets for fungal disease therapy. In addition, transnitrosylation/denitrosylation redox signaling is preserved in this fungus [145].

Although the roles played by RNS in stress and cell signaling are becoming increasingly well characterized, there are some important open questions to be answered. In murine models, NO production is essential for resistance, while overproduction seems to be associated with susceptibility [146]. However, this stoichiometry needs warrants further investigation. In mice, activated peritoneal macrophages produce NO, inhibit the conidia-to-yeast switch [147], and enhance the killing ability of macrophages [148]. In addition, iNOS KO mice infected with *P. brasiliensis* showed a poor formation of granulomas and allowed fungal dissemination with the subsequent progression of the disease [149]. The role of NO in the murine response to infection is well established, but there is some concern regarding the extrapolation of these findings to the human host. The immunohistochemistry analysis in biopsies of lesions and lymph nodes of PCM patients showed a small number of iNOS-positive macrophages around the granulomas and in multinucleated giant cells with fungi in their cytoplasm [150] However, Bordon-Gracini et al. (2012) reported that iNOS mRNA expression in human macrophages did not correlate with increases in NO production [151]. Thus, although murine models have made important contributions to determining NO functions in PCM, studies using human patients and macrophages are scarce, and it is difficult to extrapolate NO results from animal experiments for human disease.

#### 2.5.2. Thermal Regulation

Microorganisms develop defence mechanisms and altered cellular metabolism to adapt and survive environmental oscillations [152]. Among several stress defence proteins, heat shock proteins (HSPs) are fundamental in the response to environmental alterations. Initially, it was thought that HSPs were only effective in preventing protein unfolding at high temperatures [152]. Currently, HSPs have been determined to play important roles in physiological functions, and many are active in normal cells [152]. In pathogenic dimorphic fungi, such as *Paracoccidioides* spp., *H. capsulatum*, *B. dermatitidis*, *Coccidioides* spp., *Sporothrix* spp. and *Ustilago maydis*, morphological transitions are mainly associated with temperature changes [153]. Environmental modifications, such as exposure to the mammalian host immune system, also trigger the expression and activation of HSPs. This finding suggests that the role of individual HSPs is not restricted to thermal stress but is part of an overall adaptation process of survival [154].

Several HSPs have been characterized in *P. brasiliensis*. In addition to molecular studies, the immunogenic properties and biological functions of HSPs have been identified [155,156,157,158]. A transcriptomic study of *P. brasiliensis* listed 48 genes that encode molecular chaperones and their co-chaperones [159]. These genes have been classified into families corresponding to three small chaperones, nine Hsp40s, ten Hsp60s, seven Hsp70s, five Hsp90s, four Hsp100s, and ten other chaperones [159]. Heat shock proteins *HSP60*, *MDJ1* (mitochondrial HSP40), *HSP70*, *HSP82, HSP90* and *HSP104* show increased expression during the mycelium to yeast transition in *P. brasiliensis* and after thermal stress at 42 °C [58,155,156,157,160,161].

Studies investigating Hsp60 have shown its potential therapeutic use for PCM. A recent study showed that mice infected for 21 days with *P. brasiliensis* and treated with three doses of the PbHSP60 gene cloned in a plasmid (pVAX1-PB_HSP60) exhibited decreased fungal burden and inflammatory lung injury. The increase in inflammatory cytokines (INF-g, TNF, IL-6 and IL-17) and the decrease in IL-10 levels in treated mice corroborated the data observed for fungal load and histopathology [162]. In addition, mice immunized with *P. brasiliensis* Hsp60 recombinant protein showed a protective immune response and a significant reduction in fungal burden after infection with *P. brasiliensis* [163]. Although the presence of antibodies against Hsp60 appears to be insufficient to induce protection, since recombinant Hsp was recognized by sera from PCM patients [164,165], mice previously treated with monoclonal antibodies (mAbs) generated against Hsp60 from *Histoplasma capsulatum* showed a decrease in pulmonary fungal load after challenge with *P. lutzii*. The protective effects induced by mAbs were partially due to an increase in phagocytosis exhibited by *P. lutzii* yeast cells opsonized with these mAbs [166].

Hsp70 showed a high identity (89.2%) with the homologous sequence identified in *H. capsulatum*. Immunoblotting with serum from infected patients and the monoclonal antibody against *H. capsulatum* Hsp80 identified a band of 87 kDa. In addition to the aberrant molecular weight, direct amino acid sequencing demonstrated that the protein was an Hsp70 [167]. Hsp60 and Hsp70 proteins are immunogenic and may be potential candidates for immunotherapy, as they are major targets of the host immune response during infections [152].

Hsp90 protein also regulates the proliferation and adaptation of *Paracoccidioides* spp. during environmental alterations, including interaction with the host and oxidative damage [137,168,169]. The inhibition of Hsp90 by geldanamycin (GDA) impairs yeast proliferation but has no effect on mycelial development. Additionally, GDA prevented mycelial-to-yeast differentiation through a mechanism partially dependent on calcineurin. Notably, ROS levels did not change in GDA-treated yeast or mycelia incubated at 37 °C, suggesting that Hsp90 plays different roles under normal and thermal stress conditions [170]. Furthermore, monoclonal antibodies (mAbs) against Hsp90 protein successfully opsonized *P. brasiliensis* yeast cells in co-incubations with J774.16 murine macrophage cells, implying that this mAb could act as the basis for potential immunotherapy for PCM, since no cross-reactivity with mammalian chaperones was detected [171]. Although this class of proteins is highly conserved, several studies have demonstrated its importance to fungal signaling pathways to survive, in addition to altering the host immune response, consequently altering fungal fate [171,172,173]. Thus, selective inhibitors for fungal hsp90 have been developed with acceptable therapeutic indices for the treatment of invasive fungal infections [174].

#### 2.5.3. Hypoxia

During the infectious process, oxygen is not always available to *Paracoccidioides* cells. Although there is growing understanding of the microenvironmental conditions fungal pathogens encounter as they colonize their host, little is known about how *Paracoccidioides* respond to oxygen limitation, particularly in hypoxic conditions found in host tissues. The persistence of *P. brasiliensis* in tissues leads to granuloma formation in the presence of activated cells (mainly macrophages and T helper 1-Th1 cells) [175]. Granuloma inhibits fungal growth and replication by several mechanisms, including restricting access to oxygen and nutrients and exposing the fungi to acidic pH and other immune effectors [176,177]. On the other hand, the fungus within the granuloma may also benefit from this isolated microenvironment. Granuloma can provide shelter against destruction by the host and be a source of surviving pathogens that emerge in latent infection reactivation [178], as in PCM [148].

In recent years, accumulating evidence has indicated that pathogenic fungi must adapt quickly to changes in oxygen levels during infection [178,179,180,181,182]. Human pathogenic fungi respond to hypoxia by activating sterol responsive element binding proteins (SREBPs) or Upc2 and Em22 [183]. SREBP was characterized in *P. lutzii* (PlSrbA) in response to hypoxia [182]. Metabolic and respiratory changes are essential to re-establishing oxygen homeostasis under hypoxic conditions. A study investigating *P. lutzii* demonstrated an increase in proteins involved in glycolysis/gluconeogenesis and GABA shunting in 24 h under hypoxia. However, proteins associated with the TCA cycle were reduced [182]. Recently, a haem protein (fungoglobin-FglA) responsive to hypoxia has been characterized in *P. brasiliensis*. The knockdown of FglA in *Paracoccidioides* causes growth deficiency under hypoxic conditions, demonstrating the importance of this gene for fungal adaptation to low oxygen concentrations [184].

Although few studies have investigated the behavior of the *Paracoccidioides* genus under conditions of hypoxia, tolerance to low oxygen concentrations has been studied in several other pathogenic fungi, including *Aspergillus fumigatus*, *Cryptococcus neoformans*, *Saccharomyces cerevisiae*, *Schizosaccharomyces pombe*, *Candida albicans*, *Blastocladiella emersonii,* and in the bacteria *Mycobacterium tuberculosis* [178,179,180,181,185]. In *C. albicans*, hypoxia appears to induce short- and long-term transcriptional responses. Recently, it was shown that the response to hypoxia involves the positive regulation of several pathways: glycolysis from Tye7 [186], the metabolism of unsaturated fatty acids from Efg1 [187], sterol biosynthesis from Upc2 [188], and the expression of cell wall genes and proteins [187]. Pradhan et al. (2018) showed that the cAMP-protein kinase A (PKA) signaling pathway mediates hypoxia-induced β-glucan masking, thereby attenuating phagocytic recognition, uptake, and cytokine responses in *C. albicans* [189]. This phenotype is probably important in the context of immune detection of the fungus and elimination during infection.

There are still no data available on the level and conditions of tissue hypoxia in granulomas formed in PCM, but based on comparisons with other related fungi, we can conjecture how the fungus persists in the host. *Paracoccidioides* typically cause chronic disease, and most patients develop the disease several years after contact with the fungus. The mechanisms that enable fungi to remain latent and viable inside the host for extended periods have not been elucidated to date. Naturally, such mechanisms merit further clarification. There are numerous questions to answer in this field.

## 3. Genetic Aspects of the Genus *Paracoccidioides*

Interest in genetic studies on fungi of the genus *Paracoccidioides* is increasing. Evidence of recombination in *Paracoccidioides* by potential sexual reproduction was presented by Matute et al. (2006); however, the teleomorphic or sexual form of the fungus has not been determined to date [2]. Indeed, there is evidence for gene exchange between *P. brasiliensis* and *P. americana* [190]. The presence of two MAT1 (mating type locus) genes was identified in 71 isolates of *P. brasiliensis*, confirming the sexual capacity of the fungus. Two heterothallic groups were identified, one possessing the α-box gene (MAT1-1) and another possessing the HMG gene (MAT 1-2). The distribution of these genes was 1:1 among the isolates studied. Although the MAT 1-2 form was identified in the isolates Pb18 (*P. brasiliensis*) and Pb03 (*P. americana*) and the form MAT1-1 was observed in *P. lutzii* Pb01 [191], another study with isolates Pb01, Pb73, Pb2, EE, Pb03, and 3171 showed evidence that these isolates are heterothallic, while the isolates Pb18, 7455, and 133 are homothallic [192].

Interest in sexual reproduction in pathogenic fungi extends beyond basic biology. This research may obtain important insight into virulence. Indeed, there are several links between sexual reproduction mode and virulence in fungi. For instance, *C. neoformans* produces spores and titan cells after sexual reproduction. Another well-documented phenomenon is the change in ploidy from a haploid to octoploid profile in this fungus [193]. Though research has been inconclusive, there is some evidence suggesting that sexuality plays a role in Mucorales virulence [194], while in *Ustilago, maydis,* the ability to produce disease in plants relies on mating [195]. In clinical samples from patients with HIV, Damasceno et al. (2019) found different ratios of mating types for *H. capsulatum* and that coinfection with different genotypes led to recombination and could affect the course of the disease, ultimately increasing the risk of disseminated histoplasmosis [196]. Regarding the role played by mating in *Paracoccidioides* virulence, there are gaps in the research that still require answers.

The first study to evaluate genetic aspects of the genus *Paracoccidioides* demonstrated that the conidia are uninucleate, becoming multinucleated as soon as differentiation for yeasts is initiated [197]. Another approach using pulsed-field gel electrophoresis (PFGE) to separate chromosomes and labelling with 4′,6′-diamino-2-phenyl-indole (DAPI) to quantify nucleotide fluorescence suggested that the genome of *Paracoccidioides* (isolates B-339 and 113) would have four chromosomes and would probably be diploid [198]. A subsequent karyotype study, using similar methods, evaluated 12 isolates of *Paracoccidioides* and suggested that there should be four or five chromosomes and a general diploid status. However, four of the isolates were considered haploid [199]. Another study on ploidy using flow cytometry with synchronous *Paracoccidioides* yeasts (ATCC 60855) suggested that the fungus should be haploid [200].

More recently, the whole genomic sequencing of *P. brasiliensis* (Pb18 and Pb03 isolates) and *P. lutzii* (Pb01 isolate) using the Sanger method confirmed that these fungi have five chromosomes [201]. The genome of the *P. brasiliensis* isolate Pb18 has 30 Mb with 8390 genes, while the genome of *P. lutzii* has 32.93 Mb containing 8826 genes [202]. The first mitochondrial genome assemblies of *P. brasiliensis* have 71,334 bp [203], while that of *P. americana* (Pb03) has 75 kb [201]. A more recent assembly constructed using MinION sequencing indicated substantial differences in the genome sizes of *P. brasiliensis* (117,664 bp) and *P. americana* (112,887 bp) regarding the first assemblies [204]. All these genetic studies have contributed to a better understanding of the biology of the fungus, but advances still need to be accomplished in the basic genetics of the *Paracoccidioides* genus. The progress in this field will allow us to set experiments in order to respond to important questions such as whether a specific protein is a bona fide virulence factor.

## 4. State of the Art in Research on Microorganisms that Are Difficult to Genetically Manipulate

In recent years, research attempting to characterize the process of establishment and progression of the disease has been conducted with severe limitations due to the lack of effective biotechnological tools or strategies for the genetic manipulation of fungi of the genus *Paracoccidioides* [87,168,205]. For specific deletion, insertion, or gene replacement strategies to succeed, the target cell must use the homologous recombination (HR) system to repair damage inflicted on DNA. However, many eukaryotic cells do not make preferential use of this system to repair DNA double-strand breaks, with such cells preferentially employing nonhomologous end-joining repair (NHEJ) [206]. This preference has forced researchers to utilize alternative methods, such as the insertion of a T-DNA via *Agrobacterium tumefaciens*-mediated transformation [207].

In 2004, Leal et al. described, for the first time, the procedure for transformation mediated by *A. tumefaciens* in *P. brasiliensis* [208]. The technique proved to be efficient for fungi transformation when using plasmids driven by the *Neurospora crassa* promoter; however the study was unable to determine the T-DNA insertion site. The following year, the team of Soares et al. reported the possibility of the transformation of yeasts of *P. lutzii* using electroporation [209]. This technique proved to be of low efficiency, since it generated few transformants and a high mitotic instability, a phenomenon that was attributed to the presence of multiple nuclei in the fungus. Both studies have concluded that promoters of *Aspergillus nidulans* are not effective in controlling the expression in fungi of the genus *Paracoccidioides*.

Although interest in gene silencing has grown in the field of medical mycology [210], few researchers have used anti-sense RNA technology to silence genes in *Paracoccidioides* (Table 1).

In other fungi, which are also difficult to genetically manipulate, these problems were circumvented by the generation of a mutant *ΔKU70* strain resembling the wild-type (quasi-wild type) fungus that could therefore be used as the background for subsequent rounds of gene knockout [221,222,223,224]. For the genetic manipulation of four fungi of the genus *Aspergillus*, including the pathogen *A. fumigatus*, the non-homologous end-joining (NHEJ) pathway, which is controlled by the Ku70, Ku80, and DNA ligase IV proteins, was blocked. This strategy significantly increased the rate of homologous recombination events [225,226]. Ultimately, a deficiency linked to one of these three proteins leads to the inability to use the NHEJ pathway and increases the resection of damaged DNA ends, thereby inducing increased homologous recombination [227]. In *S. cerevisiae*, the integration of T-DNA by *A. tumefaciens* is attributed to NHEJ, which appears to be the preferred pathway in many fungi [228], including *H. capsulatum* [229].

After a successful effort to construct a *ΔKU70* strain in the pathway of NHEJ repair in fungi of the genus *Aspergillus*, several other microorganisms were the target of the technique, revealing the strategic potential for molecular studies in *N. crassa*, *Coprinopsis cinerea*, *Pichia pastoris*, *Candida guilliermondii*, *S. pombe*, *Metarhizium robertsii,* and *Toxoplasma gondii* [222,223,224,230,231,232,233,234].

More recently, researchers have successfully produced mutants with loss-of-function for genes related to zinc metabolism in the *B. dermatitidis* using CRISPR/Cas9 technology associated with gene transfer via *Agrobacterium*. The efficiency of this procedure was variable but notably high [235]. The CRISPR/Cas9 system was also successfully employed in the absence of *Agrobacterium* to modify the fungi *Cryptococcus neoformans* [236] and *A. fumigatus* [237]. Notably, the CRISPR/Cas9 system functions by inducing double-stranded DNA breaks to activate one of the two cellular DNA repair pathways—that is, NHEJ and HR [238,239].

## 5. Conclusions

PCM is a neglected disease for which cases are still underreported. The disease causes substantial morbidity and the early diagnosis of PCM remains a challenge. The main clinical features of this disease include mucocutaneous ulcers, lymphoid tissue commitment, and renal complications. The fate of PCM depends on the host’s immunological mechanisms and the response characteristics of the pathogen to the hostile environment. There are many open questions regarding fungal biology, some of which we discussed in this review. Once the main strategy to investigate potential virulence factors relies on the ability to test the performance of the microorganism in causing disease with and without the gene under investigation, the major limitation to research on virulence factors becomes the difficulty of genetically manipulating the fungus.

## Figures and Tables

**Table 1:** Genetic studies of *Paracoccidioides* published up to 2020.

Strain or Isolate	*A. tumefaciens*	Vector	Gene	Ref.
ATCC 6085	GV3101	pAD1625	HPH^+^	[208]
ATCC 6085	LBA1100	pUR5750	GFP^+^	[211]
ATCC 6085	LBA1100	pUR5750	CDC42^−^	[212]
ATCC 6085	LBA1100	pUR5750	HAD32^−^	[213]
ATCC 6085	LBA1100	pUR5750	AOX^−^	[214]
Pb339	LBA1100	pUR5750	GP43^−^	[92]
ATCC 6085	LBA1100	pUR5750	HSP90^−^	[137]
Pb339	LBA1100	pUR5750	P27^−^	[215]
Pb339	LBA1100	pUR5750	RBT5^−^	[216]
Pb18	EHA105	pCAMBIA-0380	ShBLE^+^	[217]
Pb01	LBA1100	pUR5750	CCP^−^	[135]
Pb18	LBA1100	pUR5750	14-3-3^−^	[96]
ATCC 6085	LBA1100	pUR5750	SOD1^−^/SOD3^−^	[218]
Pb18	LBA1100	pUR5750	PCN^−^	[68]
Pb18	LBA1100	pUR5750	TUFM-	[219]
Pb18	LBA1100	pUR5750	fglA^−^	[184]
Pb18	LBA1100	pUR5750	SidA^−^	[220]

## Data Availability

Not applicable.

## References

[1] Van Dyke M.C.C.C., Teixeira M.M., Barker B.M. (2019). Fantastic yeasts and where to find them: The hidden diversity of dimorphic fungal pathogens. Curr. Opin. Microbiol..

[2] Matute D.R., McEwen J.G., Puccia R., Montes B.A., San-Blas G., Bagagli E., Rauscher J.T., Restrepo A., Morais F., Niño-Vega G. (2006). Cryptic speciation and recombination in the fungus Paracoccidioides brasiliensis as revealed by gene genealogies. Mol. Biol. Evol..

[3] Teixeira M.M., Theodoro R.C., de Carvalho M.J.A., Fernandes L., Paes H.C., Hahn R.C., Mendoza L., Bagagli E., San-Blas G., Felipe M.S.S. (2009). Phylogenetic analysis reveals a high level of speciation in the Paracoccidioides genus. Mol. Phylogenet. Evol..

[4] Martinez R. (2017). New Trends in Paracoccidioidomycosis Epidemiology. J. Fungi (Basel Switz.).

[5] Tracogna M.F., Fernández Lugo S., Gariboglio Vázquez M.L., Fernández M.S., Andriani M.E., Presti S.E., Arce V., López R., Iliovich E., Marques I.A. (2019). Características clínicas y epidemiológicas de pacientes con paracoccidioidomicosis diagnosticados en un hospital de Resistencia, Chaco. Rev. Argent. Microbiol..

[6] Conti-Díaz I.A., Calegari L.F. (1979). [Paracoccidioidomycosis in Uruguay; its status and current problems]. Bol. Oficina Sanit. Panam..

[7] Rolon P.A. (1976). [Paracoccidioidomycosis: An epidemic in the Republic of Paraguay, the center of South America]. Mycopathologia.

[8] Alva Z.B. (2002). Aspectos clínicos de la Blastomicosis sudamericana (Paracoccidioidomicosis) en el Perú. Rev. Peru. Med. Exp. Salud Publica.

[9] Fernandez T., Lazo R.F., Mera R. (1987). Prevalencia de la paracoccidioidomicosis e histoplasmosis en la cuenca del Rio Guayas. Rev. Ecuat. Hig. Med. Trop..

[10] Torrado E., Castañeda E., De la Hoz F., Restrepo A. (2000). Paracoccidioidomicocis: Definición de las áreas endémicas de Colombia. Biomédica.

[11] Salzer H.J.F.J.F., Burchard G., Cornely O.A.A., Lange C., Rolling T., Schmiedel S., Libman M., Capone D., Le T., Dalcolmo M.P.P. (2018). Diagnosis and Management of Systemic Endemic Mycoses Causing Pulmonary Disease. Respiration.

[12] López-Martínez R., Hernández-Hernández F., Méndez-Tovar L.J., Manzano-Gayosso P., Bonifaz A., Arenas R., del Padilla-Desgarennes M.C., Estrada R., Chávez G. (2014). Paracoccidioidomycosis in Mexico: Clinical and epidemiological data from 93 new cases (1972–2012). Mycoses.

[13] Onda H., Komine M., Murata S., Ohtsuki M. (2011). Letter: Imported paracoccidioidomycosis in Japan. Dermatol. Online J..

[14] Van Damme P.A., Bierenbroodspot F., Telgt D.S.C., Kwakman J.M., De Wilde P.C.M., Meis J.F.G.M. (2006). A case of imported paracoccidioidomycosis: An awkward infection in the Netherlands. Med. Mycol..

[15] Horré R., Schumacher G., Alpers K., Seitz H.M., Adler S., Lemmer K., de Hoog G.S., Schaal K.P., Tintelnot K. (2002). A case of imported paracoccidioidomycosis in a German legionnaire. Med. Mycol..

[16] Wagner G., Moertl D., Eckhardt A., Sagel U., Wrba F., Dam K., Willinger B. (2016). Chronic Paracoccidioidomycosis with adrenal involvement mimicking tuberculosis—A case report from Austria. Med. Mycol. Case Rep..

[17] Shikanai-Yasuda M.A., Mendes R.P., Colombo A.L., de Telles F.Q., Kono A., Paniago A.M.M., Nathan A., do Valle A.C.F., Bagagli E., Benard G. (2018). II Consenso Brasileiro em Paracoccidioidomicose—2017. Epidemiol. Serviços Saúde.

[18] Borges-Walmsley M.I.I., Chen D., Shu X., Walmsley A.R. (2002). The pathobiology of Paracoccidioides brasiliensis. Trends Microbiol..

[19] Restrepo A., Benard G., de Castro C., Agudelo C., Tobón A. (2008). Pulmonary Paracoccidioidomycosis. Semin. Respir. Crit. Care Med..

[20] Mamoni R.L., Blotta M.H.S.L. (2006). Flow-cytometric analysis of cytokine production in human paracoccidioidomycosis. Cytokine.

[21] de Castro L.F., Ferreira M.C., da Silva R.M., de Blotta M.H.S.L., Longhi L.N.A., Mamoni R.L. (2013). Characterization of the immune response in human paracoccidioidomycosis. J. Infect..

[22] Felipe M.S.S., Andrade R.V., Arraes F.B.M.M., Nicola A.M., Maranhão A.Q., Torres F.A.G.G., Silva-Pereira I., Poças-Fonseca M.J., Campos E.G., Moraes L.M.P.P. (2005). Transcriptional profiles of the human pathogenic fungus Paracoccidioides brasiliensis in mycelium and yeast cells. J. Biol. Chem..

[23] Calich V.L.G., Mamoni R.L., Loures F.V. (2019). Regulatory T cells in paracoccidioidomycosis. Virulence.

[24] Stover E.P., Schär G., Clemons K.V., Stevens D.A., Feldman D. (1986). Estradiol-binding proteins from mycelial and yeast-form cultures of Paracoccidioides brasiliensis. Infect. Immun..

[25] Shankar J., Restrepo A., Clemons K.V., Stevens D.A. (2011). Hormones and the resistance of women to paracoccidioidomycosis. Clin. Microbiol. Rev..

[26] Aristizábal B.H.H., Clemons K.V.V., Cock A.M., Restrepo A., Stevens D.A. (2002). Experimental Paracoccidioides brasiliensis infection in mice: Influence of the hormonal status of the host on tissue responses. Med. Mycol..

[27] dos Santos R.P., Maia A.L., Goldani L.Z. (2004). Paracoccidioidomycosis in a woman with idiopathic hirsutism. Mycopathologia.

[28] Caixeta C.A., de Carli M.L., Ribeiro Júnior N.V., Sperandio F.F., Nonogaki S., Nogueira D.A., Pereira A.A.C., Hanemann J.A.C. (2018). Estrogen Receptor-α Correlates with Higher Fungal Cell Number in Oral Paracoccidioidomycosis in Women. Mycopathologia.

[29] Kurai H., Ohmagari N., Ito K., Kawamura I., Suzuki J., Hadano Y., Endo M., Iida Y., Okinaka K., Kamei K. (2012). A Case of Oral Paracoccidioidomycosis Suspected to be Pharyngeal Cancer. Med. Mycol. J..

[30] Steinbrück K., Fernandes R. (2018). Biliary Paracoccidioidomycosis: An Unusual Infection Simulating Malignant Neoplasm. Ann. Hepatol..

[31] Garbim B.B., D’Ávila L., Rigatto S.Z.P., da Quadros K.R.S., Belangero V.M.S., de Oliveira R.B., D’Ávila L., Rigatto S.Z.P., da Quadros K.R.S., Belangero V.M.S. (2017). Hypercalcemia in children: Three cases report with unusual clinical presentations. J. Bras. Nefrol..

[32] Bernardes Filho F., Sgarbi I., Flávia da Silva Domingos S., Sampaio R.C.R., Queiroz R.M., Fonseca S.N.S., Hay R.J., Towersey L. (2018). Acute paracoccidioidomycosis with duodenal and cutaneous involvement and obstructive jaundice. Med. Mycol. Case Rep..

[33] de Almeida Jr. J., Peçanha-Pietrobom P., Colombo A. (2018). Paracoccidioidomycosis in Immunocompromised Patients: A Literature Review. J. Fungi.

[34] Barreto M.M., Marchiori E., Amorim V.B., Zanetti G., Takayassu T.C., Escuissato D.L., Souza A.S., Rodrigues R.S. (2012). Thoracic Paracoccidioidomycosis: Radiographic and CT findings. Radiographics.

[35] Pinheiro B.G., Hahn R.C., de Camargo Z.P., Rodrigues A.M. (2020). Molecular tools for detection and identification of paracoccidioides species: Current status and future perspectives. J. Fungi.

[36] do Carmo Silva L., de Oliveira A.A., de Souza D.R., Barbosa K.L.B., Freitas e Silva K.S., Carvalho Júnior M.A.B., Rocha O.B., Lima R.M., Santos T.G., de Almeida Soares C.M. (2020). Overview of Antifungal Drugs against Paracoccidioidomycosis: How Do We Start, Where Are We, and Where Are We Going?. J. Fungi.

[37] Queiroz-Telles F., Fahal A.H., Falci D.R., Caceres D.H., Chiller T., Pasqualotto A.C. (2017). Neglected endemic mycoses. Lancet Infect. Dis..

[38] Bickford D., Lohman D.J., Sodhi N.S., Ng P.K.L., Meier R., Winker K., Ingram K.K., Das I. (2007). Cryptic species as a window on diversity and conservation. Trends Ecol. Evol..

[39] Theodoro R.C., Teixeira M.D.M., Felipe M.S.S., Paduan K.D.S., Ribolla P.M., San-Blas G., Bagagli E. (2012). Genus paracoccidioides: Species recognition and biogeographic aspects. PLoS ONE.

[40] Turissini D.A., Gomez O.M., Teixeira M.M., McEwen J.G., Matute D.R. (2017). Species boundaries in the human pathogen Paracoccidioides. Fungal Genet. Biol..

[41] de Macedo P.M., Almeida-Paes R., Freitas D.F.S., Brito-Santos F., Figueiredo-Carvalho M.H.G., de Almeida Soares J.C., Freitas A.D., Zancopé-Oliveira R.M., do Valle A.C.F. (2017). Hepatic Disease with Portal Hypertension and Acute Juvenile Paracoccidioidomycosis: A Report of Two Cases and Literature Review. Mycopathologia.

[42] Hahn R.C., Rodrigues A.M., Della Terra P.P., Nery A.F., Hoffmann-Santos H.D., Góis H.M., Fontes C.J.F., de Camargo Z.P., Terra P.P.D., Nery A.F. (2019). Clinical and epidemiological features of paracoccidioidomycosis due to paracoccidioides lutzii. PLoS Negl. Trop. Dis..

[43] Vaz C.A., Singer-Vermes L.M., Calich V.L. (1998). Comparative studies on the antibody repertoire produced by susceptible and resistant mice to virulent and nonvirulent Paracoccidioides brasiliensis isolates. Am. J. Trop. Med. Hyg..

[44] Kurokawa C.S., Lopes C.R., Sugizaki M.F., Kuramae E.E., Franco M.F., Peraçoli M.T.S. (2005). Virulence profile of ten Paracoccidioides brasiliensis isolates: Association with morphologic and genetic patterns. Rev. Inst. Med. Trop. Sao Paulo.

[45] Castilho D.G., Chaves A.F.A., Xander P., Zelanis A., Kitano E.S., Serrano S.M.T., Tashima A.K., Batista W.L. (2014). Exploring potential virulence regulators in Paracoccidioides brasiliensis isolates of varying virulence through quantitative proteomics. J. Proteome Res..

[46] Headley S.A., Pretto-Giordano L.G., Di Santis G.W., Gomes L.A., Macagnan R., da Nóbrega D.F., Leite K.M., de Alcântara B.K., Itano E.N., Alfieri A.A. (2017). Paracoccidioides brasiliensis-associated dermatitis and lymphadenitis in a dog. Mycopathologia.

[47] Bagagli E., Bosco S.M.G., Theodoro R.C., Franco M. (2006). Phylogenetic and evolutionary aspects of Paracoccidioides brasiliensis reveal a long coexistence with animal hosts that explain several biological features of the pathogen. Infect. Genet. Evol..

[48] Vidal M.S., de Melo N.T., Garcia N.M., Del Negro G.M., de Assis C.M., Heins-Vaccari E.M., Naiff R.D., Mendes R.P., da Silva Lacaz C. (1995). Paracoccidioides brasiliensis. A mycologic and immunochemical study of a sample isolated from an armadillo (*Dasipus novencinctus*). Rev. Inst. Med. Trop. Sao Paulo.

[49] Hrycyk M.F., Garcia Garces H., de Bosco S.M.G., de Oliveira S.L., Marques S.A., Bagagli E. (2018). Ecology of Paracoccidioides brasiliensis, P. lutzii and related species: Infection in armadillos, soil occurrence and mycological aspects. Med. Mycol..

[50] Storrs E.E., Walsh G.P., Burchfield H.P., Binford C.H. (1974). Leprosy in the armadillo: New model for biomedical research. Science.

[51] Nemecek J.C., Wüthrich M., Klein B.S. (2006). Global Control of Dimorphism and Virulence in Fungi. Science.

[52] Bocca A.L., Amaral A.C., Teixeira M.M., Sato P.K., Shikanai-Yasuda M.A., Soares Felipe M.S. (2013). Paracoccidioidomycosis: Eco-epidemiology, taxonomy and clinical and therapeutic issues. Future Microbiol..

[53] da Lacaz C.S., Vidal M.S., Heins-Vaccari E.M., de Melo N.T., Del Negro G.M., Arriagada G.L., dos Freitas R.S. (1999). Paracoccidioides brasiliensis. A mycologic and immunochemical study of two strains. Rev. Inst. Med. Trop. Sao Paulo.

[54] Sestari S.J., Brito W.A., Neves B.J., Soares C.M.A., Salem-Izacc S.M. (2018). Inhibition of protein kinase A affects Paracoccidioides lutzii dimorphism. Int. J. Biol. Macromol..

[55] Chen D., Janganan T.K., Chen G., Marques E.R., Kress M.R., Goldman G.H., Walmsley A.R., Borges-Walmsley M.I. (2007). The cAMP pathway is important for controlling the morphological switch to the pathogenic yeast form of Paracoccidioides brasiliensis. Mol. Microbiol..

[56] Borges-Walmsley M.I., Walmsley A.R. (2000). Response from borges-walmsley and walmsley. Trends Microbiol..

[57] Rocha C.R.C., Schröppel K., Harcus D., Marcil A., Dignard D., Taylor B.N., Thomas D.Y., Whiteway M., Leberer E. (2001). Signaling through adenylyl cyclase is essential for hyphal growth and virulence in the pathogenic fungus Candida albicans. Mol. Biol. Cell.

[58] Goldman G.H., Dos Reis Marques E., Duarte Ribeiro D.C., de Souza Bernardes L.Â., Quiapin A.C., Vitorelli P.M., Savoldi M., Semighini C.P., De Oliveira R.C., Nunes L.R. (2003). Expressed Sequence Tag Analysis of the Human Pathogen Paracoccidioides brasiliensis Yeast Phase: Identification of Putative Homologues of Candida albicans Virulence and Pathogenicity Genes. Eukaryot. Cell.

[59] Nunes L.R., Costa de Oliveira R., Leite D.B., da Silva V.S., dos Reis Marques E., Da Silva Ferreira M.E.M.E., Ribeiro D.C.D., de Souza Bernardes L.Â.A., Goldman M.H.S., Puccia R. (2005). Transcriptome Analysis of Paracoccidioides brasiliensis Cells Undergoing Mycelium-to-Yeast Transition. Eukaryot. Cell.

[60] Araújo D.S., Pereira M., Portis I.G., dos Santos Junior A.D.C.M., Fontes W., de Sousa M.V., do Assunção L.P., Baeza L.C., Bailão A.M., Ricart C.A.O. (2019). Metabolic Peculiarities of Paracoccidioides brasiliensis Dimorphism as Demonstrated by iTRAQ Labeling Proteomics. Front. Microbiol..

[61] Bastos K.P., Bailão A.M., Borges C.L., Faria F.P., Felipe M.S.S., Silva M.G., Martins W.S., Fiúza R.B., Pereira M., Soares C.M.A. (2007). The transcriptome analysis of early morphogenesis in Paracoccidioides brasiliensis mycelium reveals novel and induced genes potentially associated to the dimorphic process. BMC Microbiol..

[62] de Oliveira A.R., Oliveira L.N., Chaves E.G.A., Weber S.S., Bailão A.M., Parente-Rocha J.A., Baeza L.C., de Almeida Soares C.M., Borges C.L. (2018). Characterization of extracellular proteins in members of the Paracoccidioides complex. Fungal Biol..

[63] Rezende T.C.V.V., Borges C.L., Magalhães A.D., de Sousa M.V., Ricart C.A.O.O., Bailão A.M., Soares C.M.A.A. (2011). A quantitative view of the morphological phases of Paracoccidioides brasiliensis using proteomics. J. Proteomics.

[64] Weber S.S., Parente A.F.A., Borges C.L., Parente J.A., Bailão A.M., de Almeida Soares C.M. (2012). Analysis of the Secretomes of Paracoccidioides Mycelia and Yeast Cells. PLoS ONE.

[65] Chaves A.F.A., Navarro M.V., Castilho D.G., Calado J.C.P., Conceição P.M., Batista W.L. (2016). A conserved dimorphism-regulating histidine kinase controls the dimorphic switching in Paracoccidioides brasiliensis. FEMS Yeast Res..

[66] da Fonseca C.A., Jesuino R.S.A., Felipe M.S.S., Cunha D.A., Brito W.A., Soares C.M.A. (2001). Two-dimensional electrophoresis and characterization of antigens from Paracoccidioides brasiliensis. Microbes Infect..

[67] Fernandes L., Paes H.C., Tavares A.H., Silva S.S., Dantas A., Soares C.M.A., Torres F.A.G., Felipe M.S.S. (2008). Transcriptional profile of ras1 and ras2 and the potential role of farnesylation in the dimorphism of the human pathogen Paracoccidioides brasiliensis. FEMS Yeast Res..

[68] Fernandes F.F., Oliveira A.F., Landgraf T.N., Cunha C., Carvalho A., Vendruscolo P.E., Gonçales R.A., Almeida F., da Silva T.A., Rodrigues F. (2017). Impact of Paracoccin Gene Silencing on Paracoccidioides brasiliensis Virulence. MBio.

[69] de Curcio J.S., Paccez J.D., Novaes E., Brock M., de Almeida Soares C.M. (2018). Cell Wall Synthesis, Development of Hyphae and Metabolic Pathways Are Processes Potentially Regulated by MicroRNAs Produced Between the Morphological Stages of Paracoccidioides brasiliensis. Front. Microbiol..

[70] Toledo M.S., Levery S.B., Straus A.H., Suzuki E., Momany M., Glushka J., Moulton J.M., Takahashi H.K. (1999). Characterization of sphingolipids from mycopathogens: Factors correlating with expression of 2-hydroxy fatty acyl (E)-Δ3-unsaturation in cerebrosides of Paracoccidioides brasiliensis and Aspergillus fumigatus. Biochemistry.

[71] Niño-Vega G.A., Munro C.A., San-Blas G., Gooday G.W., Gow N.A. (2000). Differential expression of chitin synthase genes during temperature-induced dimorphic transitions in Paracoccidioides brasiliensis. Med. Mycol..

[72] Wagener J., MacCallum D.M., Brown G.D., Gow N.A.R. (2017). Candida albicans Chitin Increases Arginase-1 Activity in Human Macrophages, with an Impact on Macrophage Antimicrobial Functions. MBio.

[73] Puccia R., Vallejo M.C., Longo L.V.G. (2016). The Cell Wall-Associated Proteins in the Dimorphic Pathogenic Species of Paracoccidioides. Curr. Protein Pept. Sci..

[74] Santos L.A., Grisolia J.C., Burger E., de Araujo Paula F.B., Dias A.L.T., Malaquias L.C.C. (2020). Virulence factors of Paracoccidioides brasiliensis as therapeutic targets: A review. Antonie Leeuwenhoek Int. J. Gen. Mol. Microbiol..

[75] Arantes T.D., Bagagli E., Niño-Vega G., San-Blas G., Theodoro R.C. (2015). Paracoccidioides brasiliensis AND Paracoccidioides lutzii, A SECRET LOVE AFFAIR. Rev. Inst. Med. Trop. Sao Paulo.

[76] Tomazett P.K., Félix C.R., Lenzi H.L., de Paula Faria F., de Almeida Soares C.M., Pereira M. (2010). 1,3-β-d-Glucan synthase of Paracoccidioides brasiliensis: Recombinant protein, expression and cytolocalization in the yeast and mycelium phases. Fungal Biol..

[77] Puccia R., Vallejo M.C., Matsuo A.L., Longo L.V.G. (2011). The Paracoccidioides Cell Wall: Past and Present Layers Toward Understanding Interaction with the Host. Front. Microbiol..

[78] Hogan L.H., Klein B.S. (1994). Altered expression of surface alpha-1,3-glucan in genetically related strains of Blastomyces dermatitidis that differ in virulence. Infect. Immun..

[79] Klein B.S., Tebbets B. (2007). Dimorphism and virulence in fungi. Curr. Opin. Microbiol..

[80] Rappleye C.A., Eissenberg L.G., Goldman W.E. (2007). Histoplasma capsulatum alpha-(1,3)-glucan blocks innate immune recognition by the beta-glucan receptor. Proc. Natl. Acad. Sci. USA.

[81] Vieira I.R., Fernandes R.K., Rodrigues D.R., Gorgulho C.M., Kaneno R., Soares Â.M.V.C. (2018). TLR9 stimulation induces increase in fungicidal activity of human dendritic cells challenged with Paracoccidioides brasiliensis. Med. Mycol..

[82] Gow N.A.R., Yadav B. (2017). Microbe profile: Candida albicans: A shape-changing, opportunistic pathogenic fungus of humans. Microbiology (UK).

[83] Ricci-Azevedo R., Gonçales R.A., Roque-Barreira M.C., Girard D. (2018). Human neutrophils are targets to paracoccin, a lectin expressed by Paracoccidioides brasiliensis. Inflamm. Res..

[84] Longo L.V.G., da Cunha J.P.C., Sobreira T.J.P., Puccia R. (2014). Proteome of cell wall-extracts from pathogenic Paracoccidioides brasiliensis: Comparison among morphological phases, isolates, and reported fungal extracellular vesicle proteins. EuPA Open Proteom..

[85] Sironi M., Cagliani R., Forni D., Clerici M. (2015). Evolutionary insights into host–pathogen interactions from mammalian sequence data. Nat. Rev. Genet..

[86] Franco M., Bagagli E., Scapolio S., da Silva Lacaz C. (2000). A critical analysis of isolation of Paracoccidioides brasiliensis from soil. Med. Mycol..

[87] Tavares A.H., Silva S.S., Bernardes V.V., Maranhão A.Q., Kyaw C.M., Poças-Fonseca M., Silva-Pereira I. (2005). Virulence insights from the Paracoccidioides brasiliensis transcriptome. Genet. Mol. Res..

[88] Cross A.S. (2008). What is a virulence factor?. Crit. Care.

[89] Dunsmore S.E., Rannels D.E. (1996). Extracellular matrix biology in the lung. Am. J. Physiol. Cell. Mol. Physiol..

[90] Mendes-Giannini M.J.S., Andreotti P.F., Vincenzi L.R., da Silva J.L.M., Lenzi H.L., Benard G., Zancopé-Oliveira R., de Matos Guedes H.L., Soares C.P. (2006). Binding of extracellular matrix proteins to Paracoccidioides brasiliensis. Microbes Infect..

[91] Barbosa M.S., Bao S.N., Andreotti P.F., De Faria F.P., Felipe M.S.S., dos Santos Feitosa L., Mendes-Giannini M.J.S., de Almeida Soares C.M., Báo S.N., Andreotti P.F. (2006). Glyceraldehyde-3-phosphate dehydrogenase of Paracoccidioides brasiliensis is a cell surface protein involved in fungal adhesion to extracellular matrix proteins and interaction with cells. Infect. Immun..

[92] Torres I., Hernandez O., Tamayo D., Muñoz J.F., Leitão N.P., García A.M., Restrepo A., Puccia R., McEwen J.G. (2013). Inhibition of PbGP43 expression may suggest that gp43 is a virulence factor in Paracoccidioides brasiliensis. PLoS ONE.

[93] Vicentini A.P., Moraes J.Z., Gesztesi J.-L., Franco M.F., de Souza W., Lopes J.D. (1997). Laminin-binding epitope on gp43 from Paracoccidioides brasiliensis is recognized by a monoclonal antibody raised against Staphylococcus aureus laminin receptor. Med. Mycol..

[94] Gesztesi J.L., Puccia R., Travassos L.R., Vicentini A.P., De Moraes J.Z., Franco M.F., Lopes J.D. (1996). Monoclonal antibodies against the 43,000 Da glycoprotein from Paracoccidioides brasiliensis modulate laminin-mediated fungal adhesion to epithelial cells and pathogenesis. Hybridoma.

[95] Donofrio F.C., Calil A.C.A., Miranda E.T., Almeida A.M.F., Benard G., Soares C.P.M.d.A.P., Veloso S.N., Soares C.P.M.d.A.P., Mendes Giannini M.J.S., De Almeida Soares C.M. (2009). Enolase from Paracoccidioides brasiliensis: Isolation and identification as a fibronectin-binding protein. J. Med. Microbiol..

[96] Marcos C.M., de Silva J.F., ds Oliveira H.C., de Assato P.A., de Singulani J.L., Lopez A.M., Tamayo D.P., Hernandez-Ruiz O., McEwen J.G., Mendes-Giannini M.J.S. (2016). Decreased expression of 14-3-3 in Paracoccidioides brasiliensis confirms its involvement in fungal pathogenesis. Virulence.

[97] Nogueira S.V., Fonseca F.L., Rodrigues M.L., Mundodi V., Abi-Chacra E.A., Winters M.S., Alderete J.F., Soares C.M.d.A., De Almeida Soares C.M. (2010). Paracoccidioides brasiliensis Enolase Is a Surface Protein That Binds Plasminogen and Mediates Interaction of Yeast Forms with Host Cells. Infect. Immun..

[98] Chaves E., Weber S., Báo S., Pereira L., Bailão A., Borges C., Soares C. (2015). Analysis of Paracoccidioides secreted proteins reveals fructose 1,6-bisphosphate aldolase as a plasminogen-binding protein. BMC Microbiol..

[99] de Fatima da Silva J., Vicentim J., de Oliveira H.C., Marcos C.M., Assato P.A., Andreotti P.F., da Silva J.L.M., Soares C.P., Benard G., Almeida A.M.F. (2015). Influence of the Paracoccidioides brasiliensis14-3-3 and gp43 proteins on the induction of apoptosis in A549 epithelial cells. Mem. Inst. Oswaldo Cruz.

[100] Hanna S.A., Monteiro da Silva J.L., Giannini M.J.S.M. (2000). Adherence and intracellular parasitism of Paracoccidioides brasiliensis in Vero cells. Microbes Infect..

[101] de Oliveira H.C., da Silva J.D.F., Scorzoni L., Marcos C.M., Rossi S.A., de Paula e Silva A.C.A., Assato P.A., da Silva R.A.M., Fusco-Almeida A.M., Mendes-Giannini M.J.S. (2015). Importance of adhesins in virulence of *Paracoccidioides* spp.. Front. Microbiol..

[102] Parente J.A., Costa M., Pereira M., de Almeida Soares C.M. (2005). Transcriptome overview of Paracoccidioides brasiliensis proteases. Genet. Mol. Res..

[103] Tacco B.A.C.D.A., Parente J.A., Barbosa M.S., Báo S.N., Gsóes T.D.S., Pereira M., Soares C.M.D.A. (2009). Characterization of a secreted aspartyl protease of the fungal pathogen Paracoccidioides brasiliensis. Med. Mycol..

[104] Castilho D.G., Chaves A.F.A., Navarro M.V., Conceição P.M., Ferreira K.S., da Silva L.S., Xander P., Batista W.L. (2018). Secreted aspartyl proteinase (PbSap) contributes to the virulence of Paracoccidioides brasiliensis infection. PLoS Negl. Trop. Dis..

[105] Parente J.A., Salem-Izacc S.M., Santana J.M., Pereira M., Borges C.L., Bailão A.M., Soares C.M. (2010). A secreted serine protease of Paracoccidioides brasiliensis and its interactions with fungal proteins. BMC Microbiol..

[106] de Oliveira P., Juliano M.A., Tanaka A.S., Carmona A.K., dos Santos S.M.B., de Barros B.C.S.C., Maza P.K., Puccia R., Suzuki E. (2017). Paracoccidioides brasiliensis induces cytokine secretion in epithelial cells in a protease-activated receptor-dependent (PAR) manner. Med. Microbiol. Immunol..

[107] Bailão A.M., Shrank A., Borges C.L., Parente J.A., Dutra V., Felipe M.S.S., Fiúza R.B., Pereira M., Soares C.M.D.A., Soares A. (2007). The transcriptional profile of Paracoccidioides brasiliensis yeast cells is influenced by human plasma. FEMS Immunol. Med. Microbiol..

[108] Almeida-Paes R., Almeida M.A., Baeza L.C., Marmello L.A.M., de Trugilho M.R.O., Nosanchuk J.D., de Soares C.M.A., Valente R.H., Zancopé-Oliveira R.M. (2020). Beyond melanin: Proteomics reveals virulence-related proteins in paracoccidioides brasiliensis and paracoccidioides lutzii yeast cells grown in the presence of l-dihydroxyphenylalanine. J. Fungi.

[109] C P Emidio E., E Urán J.M., B R Silva L., S Dias L., Doprado M., Nosanchuk J.D., Taborda C.P. (2020). Melanin as a Virulence Factor in Different Species of Genus Paracoccidioides. J. Fungi.

[110] Da Silva M.B., Marques A.F., Nosanchuk J.D., Casadevall A., Travassos L.R., Taborda C.P. (2006). Melanin in the dimorphic fungal pathogen Paracoccidioides brasiliensis: Effects on phagocytosis, intracellular resistance and drug susceptibility. Microbes Infect..

[111] Da Silva F.C., Svidzinski T.I.E., Patussi E.V., Cardoso C.P., De Oliveira Dalalio M.M., Hernandes L. (2009). Morphologic organization of pulmonary granulomas in mice infected with Paracoccidioides brasiliensis. Am. J. Trop. Med. Hyg..

[112] Campos J.H., Soares R.P., Ribeiro K., Cronemberger Andrade A., Batista W.L., Torrecilhas A.C. (2015). Extracellular Vesicles: Role in Inflammatory Responses and Potential Uses in Vaccination in Cancer and Infectious Diseases. J. Immunol. Res..

[113] Samuel M., Bleackley M., Anderson M., Mathivanan S. (2015). Extracellular vesicles including exosomes in cross kingdom regulation: A viewpoint from plant-fungal interactions. Front. Plant Sci..

[114] Oliveira D.L., Nakayasu E.S., Joffe L.S., Guimarães A.J., Sobreira T.J.P., Nosanchuk J.D., Cordero R.J.B., Frases S., Casadevall A., Almeida I.C. (2010). Characterization of yeast extracellular vesicles: Evidence for the participation of different pathways of cellular traffic in vesicle biogenesis. PLoS ONE.

[115] Zhao K., Bleackley M., Chisanga D., Gangoda L., Fonseka P., Liem M., Kalra H., Al Saffar H., Keerthikumar S., Ang C.-S. (2019). Extracellular vesicles secreted by Saccharomyces cerevisiae are involved in cell wall remodelling. Commun. Biol..

[116] Park Y.-D., Chen S.H., Camacho E., Casadevall A., Williamson P.R. (2020). Role of the ESCRT Pathway in Laccase Trafficking and Virulence of Cryptococcus neoformans. Infect. Immun..

[117] Albuquerque P.C., Nakayasu E.S., Rodrigues M.L., Frases S., Casadevall A., Zancope-Oliveira R.M., Almeida I.C., Nosanchuk J.D. (2008). Vesicular transport in Histoplasma capsulatum: An effective mechanism for trans-cell wall transfer of proteins and lipids in ascomycetes. Cell. Microbiol..

[118] Vallejo M.C., Matsuo A.L., Ganiko L., Medeiros L.C.S., Miranda K., Silva L.S., Freymüller-Haapalainen E., Sinigaglia-Coimbra R., Almeida I.C., Puccia R. (2011). The pathogenic fungus Paracoccidioides brasiliensis exports extracellular vesicles containing highly Immunogenic α-galactosyl epitopes. Eukaryot. Cell.

[119] Bielska E., Sisquella M.A., Aldeieg M., Birch C., O’Donoghue E.J., May R.C. (2018). Pathogen-derived extracellular vesicles mediate virulence in the fatal human pathogen Cryptococcus gattii. Nat. Commun..

[120] Rizzo J., Chaze T., Miranda K., Roberson R.W., Gorgette O., Nimrichter L., Matondo M., Latgé J.-P., Beauvais A., Rodrigues M.L. (2020). Characterization of Extracellular Vesicles Produced by Aspergillus fumigatus Protoplasts. mSphere.

[121] Rizzo J., Rodrigues M.L., Janbon G. (2020). Extracellular Vesicles in Fungi: Past, Present, and Future Perspectives. Front. Cell. Infect. Microbiol..

[122] Vallejo M.C., Nakayasu E.S., Longo L.V.G., Ganiko L., Lopes F.G., Matsuo A.L., Almeida I.C., Puccia R. (2012). Lipidomic analysis of extracellular vesicles from the pathogenic phase of Paracoccidioides brasiliensis. PLoS ONE.

[123] Vallejo M.C., Nakayasu E.S., Matsuo A.L., Sobreira T.J.P., Longo L.V.G., Ganiko L., Almeida I.C., Puccia R. (2012). Vesicle and vesicle-free extracellular proteome of paracoccidioides brasiliensis: Comparative analysis with other pathogenic fungi. J. Proteome Res..

[124] da Silva R.P., Heiss C., Black I., Azadi P., Gerlach J.Q., Travassos L.R., Joshi L., Kilcoyne M., Puccia R. (2015). Extracellular vesicles from Paracoccidioides pathogenic species transport polysaccharide and expose ligands for DC-SIGN receptors. Sci. Rep..

[125] Peres da Silva R., Longo L.G.V., da Cunha J.P.C., Sobreira T.J.P., Rodrigues M.L., Faoro H., Goldenberg S., Alves L.R., Puccia R. (2019). Comparison of the RNA Content of Extracellular Vesicles Derived from Paracoccidioides brasiliensis and Paracoccidioides lutzii. Cells.

[126] da Silva T.A., Roque-Barreira M.C., Casadevall A., Almeida F. (2016). Extracellular vesicles from Paracoccidioides brasiliensis induced M1 polarization in vitro. Sci. Rep..

[127] Shen Q., Rappleye C.A. (2020). Living Within the Macrophage: Dimorphic Fungal Pathogen Intracellular Metabolism. Front. Cell. Infect. Microbiol..

[128] García-Carnero L.C., Pérez-García L.A., Martínez-Álvarez J.A., Reyes-Martínez J.E., Mora-Montes H.M. (2018). Current trends to control fungal pathogens: Exploiting our knowledge in the host–pathogen interaction. Infect. Drug Resist..

[129] de Castro L.F., Longhi LN A., Paião M.R., da Silva Justo-Júnior A., de Jesus M.B., Blotta MH DS L., Mamoni R.L. (2018). NLRP3 inflammasome is involved in the recognition of Paracoccidioides brasiliensis by human dendritic cells and in the induction of Th17 cells. J. Infect..

[130] Brown A.J.P., Budge S., Kaloriti D., Tillmann A., Jacobsen M.D., Yin Z., Ene I.V., Bohovych I., Sandai D., Kastora S. (2014). Stress adaptation in a pathogenic fungus. J. Exp. Biol..

[131] Allen R.G., Tresini M. (2000). Oxidative stress and gene regulation. Free Radic. Biol. Med..

[132] Haniu A.E.C.J., Maricato J.T., Mathias P.P.M., Castilho D.G., Miguel R.B., Monteiro H.P., Puccia R., Batista W.L. (2013). Low Concentrations of Hydrogen Peroxide or Nitrite Induced of Paracoccidioides brasiliensis Cell Proliferation in a Ras-Dependent Manner. PLoS ONE.

[133] de Arruda Grossklaus D., Bailão A.M., Vieira Rezende T.C., Borges C.L., de Oliveira M.A.P., Parente J.A., de Almeida Soares C.M. (2013). Response to oxidative stress in Paracoccidioides yeast cells as determined by proteomic analysis. Microbes Infect..

[134] Camacho E., Niño-Vega G.A. (2017). Paracoccidioides Spp.: Virulence Factors and Immune-Evasion Strategies. Mediators Inflamm..

[135] Parente-Rocha J.A., Parente A.F.A., Baeza L.C., Bonfim S.M.R.C., Hernandez O., McEwen J.G., Bailão A.M., Taborda C.P., Borges C.L., De Almeida Soares C.M. (2015). Macrophage interaction with paracoccidioides brasiliensis yeast cells modulates fungal metabolism and generates a response to oxidative stress. PLoS ONE.

[136] Conceição P.M., Chaves A.F.A., Navarro M.V., Castilho D.G., Calado J.C.P., Haniu A.E.C.J., Xander P., Batista W.L. (2019). Cross-talk between the Ras GTPase and the Hog1 survival pathways in response to nitrosative stress in Paracoccidioides brasiliensis. Nitric Oxide Biol. Chem..

[137] Tamayo D., Muñoz J.F., Torres I., Almeida A.J., Restrepo A., McEwen J.G., Hernández O. (2013). Involvement of the 90 kDa heat shock protein during adaptation of Paracoccidioides brasiliensis to different environmental conditions. Fungal Genet. Biol..

[138] Castilho D.G., Navarro M.V., Chaves A.F.A.A., Xander P., Batista W.L. (2018). Recovery of the Paracoccidioides brasiliensis virulence after animal passage promotes changes in the antioxidant repertoire of the fungus. FEMS Yeast Res..

[139] Chaves A.F.A., Castilho D.G., Navarro M.V., Oliveira A.K., Serrano S.M.T., Tashima A.K., Batista W.L. (2017). Phosphosite-specific regulation of the oxidative-stress response of Paracoccidioides brasiliensis: A shotgun phosphoproteomic analysis. Microbes Infect..

[140] Maniscalco M., Bianco A., Mazzarella G., Motta A. (2016). Recent Advances on Nitric Oxide in the Upper Airways. Curr. Med. Chem..

[141] Gonzalez A., Restrepo A., Cano L.E. (2007). Role of iron in the nitric oxide-mediated fungicidal mechanism of IFN-gamma-activated murine macrophages against Paracoccidioides brasiliensis conidia. Rev. Inst. Med. Trop. Sao Paulo.

[142] Parente A.F.A., Naves P.E.C., Pigosso L.L., Casaletti L., McEwen J.G., Parente-Rocha J.A., Soares C.M.A. (2015). The response of Paracoccidioides spp. to nitrosative stress. Microbes Infect..

[143] Spickett C.M., Pitt A.R., Morrice N., Kolch W. (2006). Proteomic analysis of phosphorylation, oxidation and nitrosylation in signal transduction. Biochim. Biophys. Acta—Proteins Proteom..

[144] Navarro M.V., Chaves A.F.A., Castilho D.G., Casula I., Calado J.C.P., Conceição P.M., Iwai L.K., de Castro B.F., Batista W.L. (2020). Effect of Nitrosative Stress on the S-Nitroso-Proteome of Paracoccidioides brasiliensis. Front. Microbiol..

[145] Kaufmann S.H.E. (1990). Heat shock proteins and the immune response. Immunol. Today.

[146] Nascimento F.R.F., Calich V.L.G., Rodríguez D., Russo M. (2002). Dual Role for Nitric Oxide in Paracoccidioidomycosis: Essential for Resistance, but Overproduction Associated with Susceptibility. J. Immunol..

[147] Gonzalez A., De Gregori W., Velez D., Restrepo A., Cano L.E. (2000). Nitric oxide participation in the fungicidal mechanism of gamma interferon-activated murine macrophages against Paracoccidioides brasiliensis conidia. Infect. Immun..

[148] Moreira A.P., Cavassani K.A., Tristão F.S.M., Campanelli A.P., Martinez R., Rossi M.A., Silva J.S. (2008). CCR5-Dependent Regulatory T Cell Migration Mediates Fungal Survival and Severe Immunosuppression. J. Immunol..

[149] Nishikaku A.S., Molina R.F.S., Ribeiro L.C., Scavone R., Albe B.P., Cunha C.S., Burger E. (2009). Nitric oxide participation in granulomatous response induced by Paracoccidioides brasiliensis infection in mice. Med. Microbiol. Immunol..

[150] Neworal E.P.M., Altemani A., Mamoni R.L., Noronha I.L., Blotta M.H.S.L. (2003). Immunocytochemical localization of cytokines and inducible nitric oxide synthase (iNOS) in oral mucosa and lymph nodes of patients with paracoccidioidomycosis. Cytokine.

[151] Bordon-Graciani A.P., Dias-Melicio L.A., Acorci-Valério M.J., Araujo J.P., de Campos Soares A.M.V. (2012). High expression of human monocyte iNOS mRNA induced by Paracoccidioides brasiliensis is not associated with increase in NO production. Microbes Infect..

[152] Cleare L.G., Zamith-Miranda D., Nosanchuk J.D. (2017). Heat Shock Proteins in Histoplasma and Paracoccidioides. Clin. Vaccine Immunol..

[153] Sil A., Andrianopoulos A. (2015). Thermally Dimorphic Human Fungal Pathogens—Polyphyletic Pathogens with a Convergent Pathogenicity Trait. Cold Spring Harb. Perspect. Med..

[154] Burnie J.P., Carter T.L., Hodgetts S.J., Matthews R.C. (2006). Fungal heat-shock proteins in human disease. FEMS Microbiol. Rev..

[155] Batista W.L., Matsuo A.L., Ganiko L., Barros T.F., Veiga T.R., Freymüller E., Puccia R. (2006). The PbMDJ1 gene belongs to a conserved MDJ1/LON locus in thermodimorphic pathogenic fungi and encodes a heat shock protein that localizes to both the mitochondria and cell wall of Paracoccidioides brasiliensis. Eukaryot. Cell.

[156] Da Silva S.P., Borges-Walmsley M.I., Pereira I.S., De Almeida Soares C.M., Walmsley A.R., Felipe M.S.S. (1999). Differential expression of an hsp70 gene during transition from the mycelial to the infective yeast form of the human pathogenic fungus Paracoccidioides brasiliensis. Mol. Microbiol..

[157] Izacc S.M.S., Gomez F.J., Jesuino R.S.A., Fonseca C.A., Felipe M.S.S., Deepe G.S., Soares C.M.A. (2001). Molecular cloning, characterization and expression of the heat shock protein 60 gene from the human pathogenic fungus Paracoccidioides brasiliensis. Med. Mycol..

[158] Bisio L.C., Silva S.P., Pereira I.S., Xavier M.A.S., Venâncio E.J., Puccia R., Soares C.M.A., Felipe M.S.S. (2005). A new Paracoccidiodes brasiliensis 70-kDa heat shock protein reacts with sera from paracoccidioidomycosis patients. Med. Mycol..

[159] Nicola A.M., Andrade R.V., Silva-Pereira I. (2005). Molecular chaperones in the Paracoccidioides brasiliensis transcriptome. Genet. Mol. Res..

[160] Batista W.L., Barros T.F., Goldman G.H., Morais F.V., Puccia R. (2007). Identification of transcription elements in the 5’ intergenic region shared by LON and MDJ1 heat shock genes from the human pathogen Paracoccidioides brasiliensis. Evaluation of gene expression. Fungal Genet. Biol..

[161] Silva M.G., Schrank A., Bailão E.F.L.C., Bailão A.M., Borges C.L., Staats C.C., Parente J.A., Pereira M., Salem-Izacc S.M., Mendes-Giannini M.J.S. (2011). The Homeostasis of Iron, Copper, and Zinc in Paracoccidioides Brasiliensis, Cryptococcus Neoformans Var. Grubii, and Cryptococcus Gattii: A Comparative Analysis. Front. Microbiol..

[162] Souza I.E.L., Fernandes F.F., Schiavoni M.C.L., Silva C.L., Panunto-Castelo A. (2019). Therapeutic effect of DNA vaccine encoding the 60-kDa-heat shock protein from Paracoccidoides brasiliensis on experimental paracoccidioidomycosis in mice. Vaccine.

[163] De Bastos Ascenço Soares R., Gomez F.J., De Almeida Soares C.M., Deepe G.S. (2008). Vaccination with heat shock protein 60 induces a protective immune response against experimental Paracoccidioides brasiliensis pulmonary infection. Infect. Immun..

[164] Peron G., Fernandes F.F., Landgraf T.N., Martinez R., Panunto-Castelo A. (2017). Recombinant 60-kDa heat shock protein from Paracoccidioides brasiliensis: Is it a good antigen for serological diagnosis of paracoccidioidomycosis?. Braz. J. Med. Biol. Res..

[165] Cunha D.A., Zancopé-Oliveira R.M., Felipe M.S.S., Salem-Izacc S.M., Deepe G.S., Soares C.M.A. (2002). Heterologous expression, purification, and immunological reactivity of a recombinant HSP60 from Paracoccidioides brasiliensis. Clin. Diagn. Lab. Immunol..

[166] Thomaz L., Nosanchuk J.D., Rossi D.C.P., Travassos L.R., Taborda C.P. (2014). Monoclonal antibodies to heat shock protein 60 induce a protective immune response against experimental Paracoccidioides lutzii. Microbes Infect..

[167] Díez S., Gómez B.L., Restrepo A., Hay R.J., Hamilton A.J. (2002). Paracoccidioides brasiliensis 87-kilodalton antigen, a heat shock protein useful in diagnosis: Characterization, purification, and detection in biopsy material via immunohistochemistry. J. Clin. Microbiol..

[168] Nicola A.M., Andrade R.V., Dantas A.S., Andrade P.A., Arraes F.B.M., Fernandes L., Silva-Pereira I., Felipe M.S.S. (2008). The stress responsive and morphologically regulated hsp90 gene from Paracoccidioides brasiliensis is essential to cell viability. BMC Microbiol..

[169] Araújo F.S., Coelho L.M., Silva L.D.C., da Silva Neto B.R., Parente-Rocha J.A., Bailão A.M., de Oliveira C.M.A., Fernandes G.D.R., Hernández O., Ochoa J.G.M. (2016). Effects of Argentilactone on the Transcriptional Profile, Cell Wall and Oxidative Stress of Paracoccidioides spp.. PLoS Negl. Trop. Dis..

[170] Matos T.G.F., Morais F.V., Campos C.B.L. (2013). Hsp90 regulates Paracoccidioides brasiliensis proliferation and ROS levels under thermal stress and cooperates with calcineurin to control yeast to mycelium dimorphism. Med. Mycol..

[171] Moura Á.N.D., de Oliveira D.S.L., Paredes V., Rocha L.B., de Oliveira F.F.M., Lessa G.M., Riasco-Palacios J.F., Casadevall A., Albuquerque P., Felipe M.S.S. (2020). Paracoccidioides hsp90 can be found in the cell surface and is a target for antibodies with therapeutic potential. J. Fungi.

[172] Jia C., Zhang J., Zhuge Y., Xu K., Liu J., Wang J., Li L., Chu M. (2019). Synergistic effects of geldanamycin with fluconazole are associated with reactive oxygen species in Candida tropicalis resistant to azoles and amphotericin B. Free Radic. Res..

[173] Mahmoudi S., Rezaie S., Daie Ghazvini R., Hashemi S.J., Badali H., Foroumadi A., Diba K., Chowdhary A., Meis J.F., Khodavaisy S. (2019). In Vitro Interaction of Geldanamycin with Triazoles and Echinocandins Against Common and Emerging Candida Species. Mycopathologia.

[174] Huang D.S., Leblanc E.V., Shekhar-Guturja T., Robbins N., Krysan D.J., Pizarro J., Whitesell L., Cowen L.E., Brown L.E. (2020). Design and Synthesis of Fungal-Selective Resorcylate Aminopyrazole Hsp90 Inhibitors. J. Med. Chem..

[175] de Carli M.L., Miyazawa M., Nonogaki S., Shirata N.K., Oliveira D.T., Pereira A.A.C., Hanemann J.A.C. (2016). M2 macrophages and inflammatory cells in oral lesions of chronic paracoccidioidomycosis. J. Oral Pathol. Med..

[176] Tsai M.C., Chakravarty S., Zhu G., Xu J., Tanaka K., Koch C., Tufariello J.A., Flynn J.A., Chan J. (2006). Characterization of the tuberculous granuloma in murine and human lungs: Cellular composition and relative tissue oxygen tension. Cell. Microbiol..

[177] Heninger E., Hogan L.H., Karman J., Macvilay S., Hill B., Woods J.P., Sandor M. (2006). Characterization of the Histoplasma capsulatum -Induced Granuloma. J. Immunol..

[178] Chan J., Flynn J.A. (2004). The immunological aspects of latency in tuberculosis. Clin. Immunol..

[179] Willger S.D., Puttikamonkul S., Kim K.H., Burritt J.B., Grahl N., Metzler L.J., Barbuch R., Bard M., Lawrence C.B., Cramer R.A. (2008). A sterol-regulatory element binding protein is required for cell polarity, hypoxia adaptation, azole drug resistance, and virulence in Aspergillus fumigatus. PLoS Pathog..

[180] Barker B.M., Kroll K., Vödisch M., Mazurie A., Kniemeyer O., Cramer R.A. (2012). Transcriptomic and proteomic analyses of the Aspergillus fumigatus hypoxia response using an oxygen-controlled fermenter. BMC Genom..

[181] Grahl N., Cramer R.A. (2010). Regulation of hypoxia adaptation: An overlooked virulence attribute of pathogenic fungi?. Med. Mycol..

[182] Lima P.D.S., Chung D., Bailão A.M., Cramer R.A., Soares C.M.D.A. (2015). Characterization of the Paracoccidioides Hypoxia Response Reveals New Insights into Pathogenesis Mechanisms of This Important Human Pathogenic Fungus. PLoS Negl. Trop. Dis..

[183] Synnott J.M., Guida A., Mulhern-Haughey S., Higgins D.G., Butler G. (2010). Regulation of the hypoxic response in *Candida albicans*. Eukaryot. Cell.

[184] Nojosa Oliveira L., Aguiar Gonçales R., Garcia Silva M., Melo Lima R., Vieira Tomazett M., Santana de Curcio J., Domiraci Paccez J., Milhomem Cruz-Leite V.R., Rodrigues F., de Sousa Lima P. (2020). Characterization of a heme-protein responsive to hypoxia in Paracoccidioides brasiliensis. Fungal Genet. Biol..

[185] Grahl N., Shepardson K.M., Chung D., Cramer R.A. (2012). Hypoxia and fungal pathogenesis: To air or not to air?. Eukaryot. Cell.

[186] Bonhomme J., Chauvel M., Goyard S., Roux P., Rossignol T., D’Enfert C. (2011). Contribution of the glycolytic flux and hypoxia adaptation to efficient biofilm formation by Candida albicans. Mol. Microbiol..

[187] Setiadi E.R., Doedt T., Cottier F., Noffz C., Ernst J.F. (2006). Transcriptional Response of Candida albicans to Hypoxia: Linkage of Oxygen Sensing and Efg1p-regulatory Networks. J. Mol. Biol..

[188] MacPherson S., Akache B., Weber S., De Deken X., Raymond M., Turcotte B. (2005). Candida albicans zinc cluster protein Upc2p confers resistance to antifungal drugs and is an activator of ergosterol biosynthetic genes. Antimicrob. Agents Chemother..

[189] Pradhan A., Avelar G.M., Bain J.M., Childers D.S., Larcombe D.E., Netea M.G., Shekhova E., Munro C.A., Brown G.D., Erwig L.P. (2018). Hypoxia promotes immune evasion by triggering β-glucan masking on the candida albicans cell surface via mitochondrial and cAMP-protein kinase A signaling. MBio.

[190] Teixeira M.D.M., Cattana M.E., Matute D.R., Muñoz J.F., Arechavala A., Isbell K., Schipper R., Santiso G., Tracogna F., de los Ángeles Sosa M. (2020). Genomic diversity of the human pathogen Paracoccidioides across the South American continent. Fungal Genet. Biol..

[191] Li W., Metin B., White T.C., Heitman J. (2010). Organization and evolutionary trajectory of the mating type (MAT) locus in dermatophyte and dimorphic fungal pathogens. Eukaryot. Cell.

[192] Teixeira M.D.M., Theodoro R.C., Derengowski L.D.S., Nicola A.M., Bagagli E., Felipe M.S. (2013). Molecular and morphological data support the existence of a sexual cycle in species of the genus Paracoccidioides. Eukaryot. Cell.

[193] Heitman J., Carter D.A., Dyer P.S., Soll D.R. (2014). Sexual reproduction of human fungal pathogens. Cold Spring Harb. Perspect. Med..

[194] Xu W., Liang G., Peng J., Long Z., Li D., Fu M., Wang Q., Shen Y., Lv G., Mei H. (2017). The influence of the mating type on virulence of Mucor irregularis. Sci. Rep..

[195] Feldbrügge M., Kämper J., Steinberg G., Kahmann R. (2004). Regulation of mating and pathogenic development in Ustilago maydis. Curr. Opin. Microbiol..

[196] Damasceno L.S., Teixeira M.D.M., Barker B.M., Almeida M.A., Muniz M.D.M., Pizzini C.V., Mesquita J.R.L., Rodríguez-Arellanes G., Ramírez J.A., Vite-Garín T. (2019). Novel clinical and dual infection by Histoplasma capsulatum genotypes in HIV patients from Northeastern, Brazil. Sci. Rep..

[197] McEwen J.G., Restrepo B.I., Salazar M.E., Restrepo A. (1987). Nuclear staining of *Paracoccidioides brasiliensis* conidia. Med. Mycol..

[198] Cano I.M.N., Cisalpino P.S., Galindo I., Ramírez J.L., Mortara R.A., Franco da Silveria J. (1998). Electrophoretic Karyotypes and Genome Sizing of the Pathogenic Fungus Paracoccidioides brasiliensis Electrophoretic Karyotypes and Genome Sizing of the Pathogenic Fungus Paracoccidioides brasiliensis. J. Clin. Microbiol..

[199] Feitosa L.D.S., Cisalpino P.S., Machado Dos Santos M.R., Mortara R.A., Barros T.F., Morais F.V., Puccia R., Da Silveira J.F., De Camargo Z.P. (2003). Chromosomal polymorphism, syntenic relationships, and ploidy in the pathogenic fungus Paracoccidioides brasiliensis. Fungal Genet. Biol..

[200] Almeida A.J., Matute D.R., Carmona J.A., Martins M., Torres I., McEwen J.G., Restrepo A., Leão C., Ludovico P., Rodrigues F. (2007). Genome size and ploidy of Paracoccidioides brasiliensis reveals a haploid DNA content: Flow cytometry and GP43 sequence analysis. Fungal Genet. Biol..

[201] Desjardins C.A., Champion M.D., Holder J.W., Muszewska A., Goldberg J., Bailão A.M., Brigido M.M., Ferreira M.E.D.S., Garcia A.M., Grynberg M. (2011). Comparative genomic analysis of human fungal pathogens causing paracoccidioidomycosis. PLoS Genet..

[202] Muñoz J.F., Gallo J.E., Misas E., Priest M., Imamovic A., Young S., Zeng Q., Clay O.K., McEwen J.G., Cuomo C. (2014). A Genome update of the dimorphic human pathogenic fungi causing paracoccidioidomycosis. PLoS Negl. Trop. Dis..

[203] Cardoso M.A.G., Tambor J.H.M., Nobrega F.G. (2007). The mitochondrial genome from the thermal dimorphic fungusParacoccidioides brasiliensis. Yeast.

[204] Misas E., Gómez O.M., Botero V., Muñoz J.F., Teixeira M.M., Gallo J.E., Clay O.K., McEwen J.G. (2020). Updates and Comparative Analysis of the Mitochondrial Genomes of Paracoccidioides spp. Using Oxford Nanopore MinION Sequencing. Front. Microbiol..

[205] Biondo G.A., Dias-Melicio L.A., Bordon-Graciani A.P., Kurokawa C.S., de Campos Soares A.M.V. (2012). Production of leukotriene B4 by Paracoccidioides brasiliensis. Yeast.

[206] Bartlett E.J., Brissett N.C., Plocinski P., Carlberg T., Doherty A.J. (2016). Molecular basis for DNA strand displacement by NHEJ repair polymerases. Nucleic Acids Res..

[207] Shafran H., Miyara I., Eshed R., Prusky D., Sherman A. (2008). Development of new tools for studying gene function in fungi based on the Gateway system. Fungal Genet. Biol..

[208] Leal C.V., Montes B., Mesa A.C., Rua A.L., Corredor M., Restrepo A., McEwen J.G. (2004). Agrobacterium tumefaciens -mediated transformation of Paracoccidioides brasiliensis. Med. Mycol..

[209] Soares R.D.B., Velho T.F., De Moraes L.M.P., Azevedo M.O., De A. Soares C.M., Felipe M.S.S. (2005). Hygromycin B-resistance phenotype acquired in Paracoccidioides brasiliensis via plasmid DNA integration. Med. Mycol..

[210] Menino J.F., Almeida A.J., Rodrigues F. (2012). Gene Knockdown in Paracoccidioides brasiliensis Using Antisense RNA. Methods in Molecular Biology (Clifton, N.J.).

[211] Almeida A.J., Carmona J.A., Cunha C., Carvalho A., Rappleye C.A., Goldman W.E., Hooykaas P.J., Leão C., Ludovico P., Rodrigues F. (2007). Towards a molecular genetic system for the pathogenic fungus Paracoccidioides brasiliensis. Fungal Genet. Biol..

[212] Almeida A.J., Cunha C., Carmona J.A., Sampaio-Marques B., Carvalho A., Malavazi I., Steensma H.Y., Johnson D.I., Leão C., Logarinho E. (2009). Cdc42p controls yeast-cell shape and virulence of Paracoccidioides brasiliensis. Fungal Genet. Biol..

[213] Hernández O., Almeida A.J., Gonzalez A., Garcia A.M., Tamayo D., Cano L.E., Restrepo A., McEwen J.G., Hernandez O., Almeida A.J. (2010). A 32-kilodalton hydrolase plays an important role in Paracoccidioides brasiliensis adherence to host cells and influences pathogenicity. Infect. Immun..

[214] Ruiz O.H., Gonzalez A., Almeida A.J., Tamayo D., Garcia A.M., Restrepo A., McEwen J.G., Hernández Ruiz O., Gonzalez A., Almeida A.J. (2011). Alternative oxidase mediates pathogen resistance in Paracoccidioides brasiliensis infection. PLoS Negl. Trop. Dis..

[215] Torres I., Hernandez O., Tamayo D., Muñoz J.F., García A.M., Gómez B.L., Restrepo A., McEwen J.G. (2014). Paracoccidioides brasiliensis PbP27 gene: Knockdown procedures and functional characterization. FEMS Yeast Res..

[216] Bailão E.F.L.C., Parente J.A., Pigosso L.L., de Castro K.P., Fonseca F.L., Silva-Bailão M.G., Báo S.N., Bailão A.M., Rodrigues M.L., Hernandez O. (2014). Hemoglobin uptake by Paracoccidioides spp. is receptor-mediated. PLoS Negl. Trop. Dis..

[217] Goes T., Bailão E.F.L.C., Correa C.R., Bozzi A., Santos L.I., Gomes D., Soares C.M., Goes A.M. (2014). New Developments of RNAi in Paracoccidioides brasiliensis: Prospects for High-Throughput, Genome-Wide, Functional Genomics. PLoS Negl. Trop. Dis..

[218] Tamayo D., Muñoz J.F., Lopez Á., Urán M., Herrera J., Borges C.L., Restrepo Á., Soares C.M., Taborda C.P., Almeida A.J. (2016). Identification and Analysis of the Role of Superoxide Dismutases Isoforms in the Pathogenesis of *Paracoccidioides* spp.. PLoS Negl. Trop. Dis..

[219] Marcos C.M., Tamer G., de Oliveira H.C., Assato P.A., Scorzoni L., Santos C.T., de Lacorte Singulani J., de Fátima da Silva J., de Almeida R., de Paula E Silva A.C.A. (2019). Down-regulation of TUFM impairs host cell interaction and virulence by Paracoccidioides brasiliensis. Sci. Rep..

[220] Silva M.G., de Curcio J.S., Silva-Bailão M.G., Lima R.M., Tomazett M.V., de Souza A.F., Cruz-Leite V.R.M., Sbaraini N., Bailão A.M., Rodrigues F. (2020). Molecular characterization of siderophore biosynthesis in Paracoccidioides brasiliensis. IMA Fungus.

[221] Fox B.A., Falla A., Rommereim L.M., Tomita T., Gigley J.P., Mercier C., Cesbron-Delauw M.-F., Weiss L.M., Bzik D.J. (2011). Type II Toxoplasma gondii KU80 knockout strains enable functional analysis of genes required for cyst development and latent infection. Eukaryot. Cell.

[222] Bugeja H.E., Boyce K.J., Weerasinghe H., Beard S., Jeziorowski A., Pasricha S., Payne M., Schreider L., Andrianopoulos A. (2012). Tools for high efficiency genetic manipulation of the human pathogen Penicillium marneffei. Fungal Genet. Biol..

[223] Näätsaari L., Mistlberger B., Ruth C., Hajek T., Hartner F.S., Glieder A. (2012). Deletion of the Pichia pastoris KU70 homologue facilitates platform strain generation for gene expression and synthetic biology. PLoS ONE.

[224] Foureau E., Courdavault V., Rojas L.F., Dutilleul C., Simkin A.J., Crèche J., Atehortùa L., Giglioli-Guivarc’h N., Clastre M., Papon N. (2013). Efficient gene targeting in a Candida guilliermondii non-homologous end-joining pathway-deficient strain. Biotechnol. Lett..

[225] Takahashi T., Masuda T., Koyama Y. (2006). Enhanced gene targeting frequency in ku70 and ku80 disruption mutants of *Aspergillus sojae* and *Aspergillus oryzae*. Mol. Genet. Genom..

[226] Zhang J., Mao Z., Xue W., Li Y., Tang G., Wang A., Zhang Y., Wang H. (2011). Ku80 Gene is Related to Non-Homologous End-Joining and Genome Stability in Aspergillus niger. Curr. Microbiol..

[227] Kass E.M., Jasin M. (2010). Collaboration and competition between DNA double-strand break repair pathways. FEBS Lett..

[228] Michielse C.B., Hooykaas P.J.J., van den Hondel C.M.J.J., Ram A.F.J. (2005). Agrobacterium-mediated transformation as a tool for functional genomics in fungi. Curr. Genet..

[229] Kemski M.M., Stevens B., Rappleye C.A. (2012). Spectrum of T-DNA integrations for insertional mutagenesis of Histoplasma capsulatum. Fungal Biol..

[230] Colot H.V., Park G., Turner G.E., Ringelberg C., Crew C.M., Litvinkova L., Weiss R.L., Borkovich K.A., Dunlap J.C. (2006). A high-throughput gene knockout procedure for Neurospora reveals functions for multiple transcription factors. Proc. Natl. Acad. Sci. USA.

[231] Fox B., Ristuccia J.G., Gigley J.P., Bzik D.J. (2009). Efficient gene replacements in Toxoplasma gondii strains deficient for nonhomologous end joining. Eukaryot. Cell.

[232] Nakazawa T., Ando Y., Kitaaki K., Nakahori K., Kamada T. (2011). Efficient gene targeting in ΔCc.ku70 or ΔCc.lig4 mutants of the agaricomycete Coprinopsis cinerea. Fungal Genet. Biol..

[233] Fennessy D., Grallert A., Krapp A., Cokoja A., Bridge A.J., Petersen J., Patel A., Tallada V.A., Boke E., Hodgson B. (2014). Extending the Schizosaccharomyces pombe molecular genetic toolbox. PLoS ONE.

[234] Xu C., Zhang X., Qian Y., Chen X., Liu R., Zeng G., Zhao H., Fang W. (2014). A high-throughput gene disruption methodology for the entomopathogenic fungus Metarhizium robertsii. PLoS ONE.

[235] Kujoth G.C., Sullivan T.D., Merkhofer R., Lee T.-J., Wang H., Brandhorst T., Wüthrich M., Klein B.S. (2018). CRISPR/Cas9-Mediated Gene Disruption Reveals the Importance of Zinc Metabolism for Fitness of the Dimorphic Fungal Pathogen *Blastomyces dermatitidis*. MBio.

[236] Wang P. (2018). Two Distinct Approaches for CRISPR-Cas9-Mediated Gene Editing in Cryptococcus neoformans and Related Species. mSphere.

[237] Al Abdallah Q., Ge W., Fortwendel J.R. (2017). A Simple and Universal System for Gene Manipulation in Aspergillus fumigatus: In Vitro-Assembled Cas9-Guide RNA Ribonucleoproteins Coupled with Microhomology Repair Templates. mSphere.

[238] Ma Y., Zhang L., Huang X. (2014). Genome modification by CRISPR/Cas9. FEBS J..

[239] Sander J.D., Joung J.K. (2014). CRISPR-Cas systems for editing, regulating and targeting genomes. Nat. Biotechnol..

